# Computational Prediction and Analysis of Envelop Glycoprotein Epitopes of DENV-2 and DENV-3 Pakistani Isolates: A First Step towards Dengue Vaccine Development

**DOI:** 10.1371/journal.pone.0119854

**Published:** 2015-03-16

**Authors:** Hafsa Amat-ur-Rasool, Anam Saghir, Muhammad Idrees

**Affiliations:** National Center of Excellence in Molecular Biology, University of the Punjab, Lahore, Pakistan; Texas A&M Health Science Center, UNITED STATES

## Abstract

Dengue fever of tropics is a mosquito transmitted devastating disease caused by dengue virus (DENV). There is no effective vaccine available, so far, against any of its four serotypes (DENV-1, DENV-2, DENV-3, and DENV-4). There is a need for the development of preventive and therapeutic vaccines against DENV to decrease the prevalence of dengue fever, especially in Pakistan. In this research, linear and conformational B-cell epitopes of envelope glycoprotein of DENV-2 and DENV-3 (the most prevalent serotypes in Pakistan) were predicted. We used Kolaskar and Tongaonkar method for linear epitope prediction, Emini’s method for surface accessibility prediction and Karplus and Schulz’s algorithm for flexibility determination. To propose three dimensional epitopes, the E proteins for both serotypes were homology modeled by using Phyre2 V 2.0 server, and ElliPro was used for the prediction of surface epitopes on their globular structure. Total 21 and 19 linear epitopes were predicted for DENV-2 and DENV-3 Pakistani isolates respectively. Whereas, 5 and 4 discontinuous epitopes were proposed for DENV-2 and DENV-3 Pakistani isolates respectively. Moreover, the values of surface accessibility, flexibility and solvent-accessibility can be helpful in analyzing vaccines against DENV-2 and DENV-3. In conclusion, the proposed continuous and discontinuous antigenic peptides can be valuable candidates for diagnostic and therapeutics of DENV.

## Introduction

Dengue virus (DENV), an arbovirus of family flaviviridae, is responsible for dengue fever outbreaks in tropic regions of the world in past decades [1]. Female mosquitoes of the *Aedes* genus are considered the main vector for transporting dengue virus to humans through their bite [2]. It is estimated that approximately 100 million cases of dengue fever occur annually in humans [3]. This virus is responsible to cause clinical manifestations ranging from mild asymptomatic dengue fever to severe and lethal forms of illnesses recognized as dengue hemorrhagic fever (DHF) and dengue shock syndrome (DSS) [4]. Later two (DHF and DSS) are considered relatively serious threat to human health in tropical areas of the world [5]. DENV has four serotypes namely DENV-1, DENV-2, DENV-3 and DENV-4 each of which can lead to infection [6]. Each of these antigenically related viruses provides life-long immunity for that specific serotype; however, it does not give complete protection against other serotypes [7].

All four serotypes are endemic in Pakistan. Although these viruses remain present throughout the year, however during the monsoon phase i.e., between October and December, its incidence reaches its peak [8,9]. The first epidemic of DENV appeared in Karachi, Pakistan with in duration of one year i.e., between June 1994 to September 1995 [10]. It is considered that DENV came to Pakistan by means of tyres imported from endemic countries that carried infected mosquito eggs which were present at Karachi sea port [2]. In Pakistan, till now DENV is responsible for causing numerous outbreaks [11,12]. The major outbreak of dengue fever occurred in Lahore, during 2011. In this epidemic, more than 250 people were reported dead and over 12,000 people got infected according to Punjab Health Department. DENV2 and DENV3 were the most prevalent serotypes detected in the blood samples of affected patients [13].

DENV is an enveloped virion which has plus-sense single stranded RNA genome of 11.8kb in length. The genome contains non coding regions (NCR) and a single coding region which code for a total of ten individual proteins, three of which are structural proteins namely the capsid or core protein (C) having 100 amino acids, the membrane protein (M) having 75 amino acids and envelope glycoproteins (E) having 495 amino acids; and seven non-structural (NS) proteins: NS1, NS2A, NS2B, NS3, NS4A, NS4B and NS5 [5,14,15]. The glycoproteins M and E are embedded in a lipid bilayer that exists around the nucleocapsid [16]. The icosahedron DENV consists of 180 monomers of E and M protein which are arranged in a specific pattern [15,17].

Mature virion E protein is involved in creating interactions with target cells for facilitating entry of virus in it, via specific cell surface receptors [14,18,19]. Moreover, it is also involved in cellular tropism and plays an important role in the virulence of DENV [15,20]. E protein as an antigen plays a key role in stimulating immunity of the host cell, which can be used as a target in designing peptide-based vaccine [21]. Identification of B-cell epitopes (antigenic regions that stimulate B cell response) is a foremost step to propose a peptide vaccine. There are many tools available online for prediction of linear (continuous on sequence) and conformational (3D or discontinuous) antigenic epitopes [22].

In this study, we used DENV-2 and DENV-3 Pakistani isolates E protein sequences to predict and analyze linear as well as conformational B-cell antigenic epitopes, computationally. For the prediction of structure based epitopes, we predicted 3D structure of E protein via homology modeling. Moreover, the surface accessibility and flexibility of E protein is also presumed by using *in-silico* techniques.

## Materials and Methods

### Retrieval of DENV-2 and DENV-3 Envelope Protein Sequences of Pakistani isolates

No reference sequences are available in NCBI for DENV-2PK and DENV-3PK yet. To the best of our knowledge, only twenty and sixteen partial coding sequences (cds) are available for DENV-2 and DENV-3 Pakistani isolates in NCBI-nucleotide and no entry in National Center for Biotechnology Information (NCBI)-Protein Database exists till date for these circulating DENV serotypes in Pakistan. All the available nucleotide sequences were retrieved from NCBI (http://www.ncbi.nlm.nih.gov/nucleotide) and translated by using EMBOSS-Transeq tool (http://www.ebi.ac.uk/Tools/st/emboss_transeq/). The sequence for antigenic epitope determination was selected via multiple sequence alignment by using ClustalW [23].

### Linear Epitope Prediction

The online tool integrated at Immune Epitope Database Analysis Resource (IEDB) (http://tools.immuneepitope.org/tools/bcell/iedb_input) was used to determine B cell linear epitope of DENV-2 (495 aa) and DENV-3 (493 aa) sequences using the method of Kolaskar and Tongaonkar [24]. The method is based on the occurrence of amino acid residues in experimentally determined epitopes. Application of this method can predict antigenic determinants with about 75% accuracy which is better than most of the known methods [24].

### Surface Accessibility and Flexibility Prediction

The surface accessibility of E protein sequences of DENV-2 and DENV-3 was predicted by the method of Emini [25] while surface flexibility was predicted by using Karplus and Schulz’s algorithm separately [26] by using online tools available at (http://tools.immuneepitope.org/tools/bcell/iedb_input).

### Homology Modeling of DENV-2 and DENV-3 Envelope Proteins of Pakistani isolates

To predict antigenic epitopes in 3D conformation, the E proteins of DENV-2 and DENV-3 were homology modeled by using Phyre2 V 2.0 server online available at (http://www.sbg.bio.ic.ac.uk/phyre2/html/page.cgi?id=index) [27]. The server uses PSI-BLAST to find homologue templates and model the 3D structure of provided sequence accordingly.

### Structure-based Epitope Prediction

EliPro is an online tool for predicting discontinuous epitopes from 3D structures of proteins in PDB format based upon solvent-accessibility and flexibility (http://tools.immuneepitope.org/tools/ElliPro/iedb_input) [28]. The input files for DENV-2 and DENV-3 were provided in PDB format to the server separately and minimum score value was set at 0.7 while maximum distance was selected as 6 Å.

## Results and Discussion

### Target DENV Envelope Protein Sequences

Dengue virus fever is a serious problem in a Paksitan since 2007. Unavailability of vaccine against this virus has taken many precious lives. It has been reported that DENV serotype 2 and DENV serotype 3 are the major cause of this havoc. Researchers have gathered data from patient samples and submitted to NCBI, but it is very limited. As there was no reference sequence available, so we selected the best reported sequence length based on multiple sequence alignment results for DENV-2 and DENV-3 Pakistani isolates. Accession No. KF041224 (S1 Accession Number) for DENV-2 and Accession No. KF041238 (S2 Accession Number) for DENV-3 translated by EMBOSS-Transeq were used in present study.

### Continous Epitopes of E Protein of DENV-2 and DENV-3

Kolaskar and Tongaonkar’s method predicts antigenic epitopes of given sequence, based on physicochemical properties of amino acid residues that frequently occur in experimentally determined antigenic epitopes. Previously reported data appreciated this method as it gives 75% experuimental accuracy [24]. By using this method the predicted results for DENV-2, show that it’s sequence of 495 amino acids bear 21 antigenic peptides. The length of the antigenic peptides range from 6–27 amino acids with 5 octapeptides. Peptide length, their sequences and the location of the peptides along the sequence length, are given in Table 1.

**Table 1:** Predicted antigenic epitope peptides of DENV-2 Pakistani isolate.

No.	Start Position	End Position	Peptide	Peptide Length
**1**	20	33	WVDIVLEHGSCVTT	14
**2**	42	48	DFELIKT	7
**3**	53	63	PATLRKYCIEA	11
**4**	88	95	KRFVCKHS	8
**5**	113	120	IVTCAMFT	8
**6**	128	133	KIVQPE	6
**7**	137	144	YTIVVTPH	8
**8**	161	170	EIKVTPQSSI	10
**9**	194	200	NEMVLLQ	7
**10**	205	224	AWLVHRQWFLDLPLPWLPGA	20
**11**	235	241	ETLVTFK	7
**12**	247	255	KQDVVVLGS	9
**13**	275	288	GNLLFTGHLKCRLR	14
**14**	290	297	DKLQLKGM	8
**15**	305	312	KFKVVKEI	8
**16**	318	327	GTIVVRVQYE	10
**17**	331	339	SPCKIPFEI	9
**18**	344	362	KRHVLGRLITVNPIVTEKD	19
**19**	374	391	GDSYIIIGVEPGQLKLSW	18
**20**	425	451	LGGVFTSIGKALHQVFGAIYGAAFSGV	27
**21**	456	465	KILIGVVITW	10

While, the results of DENV-3 of 493 amino acids showed that it contains 19 antigenic peptides. The length of the antigenic peptides range from 7–34 amino acids with 5 octapeptides and 5 heptapeptides. Peptide length, their sequences and the location of the peptides along the sequence length, are given in Table 2.

**Table 2:** Predicted antigenic epitope peptides of DENV-3 Pakistani isolate.

No.	Start Position	End Position	Peptide	Peptide Length
**1**	18	33	ATWVDVVLEHGGCVTT	16
**2**	51	63	TQLATLRKLCIEG	13
**3**	77	84	QGEAVLPE	8
**4**	88	97	QNYVCKHTYV	10
**5**	110	124	KGSLVTCAKFQCLEP	15
**6**	126	146	EGKVVQYENLKYTVIITVHTG	21
**7**	170	176	EAILPEY	7
**8**	178	184	TLGLECS	7
**9**	211	219	FFDLPLPWT	9
**10**	233	239	ELLVTFK	7
**11**	245	253	KQEVVVLGS	9
**12**	277	286	FAGHLKCRLK	10
**13**	302	309	NTFVLKKE	8
**14**	316	323	GTILIKVE	8
**15**	328	335	DAPCKIPF	8
**16**	351	358	TANPVVTK	8
**17**	374	380	SNIVIGI	7
**18**	423	429	VGGVLNS	7
**19**	431	464	GKMVHQIFGSAYTALFSGVSWVMKIGIGVLLTWI	34

**Fig 1:**
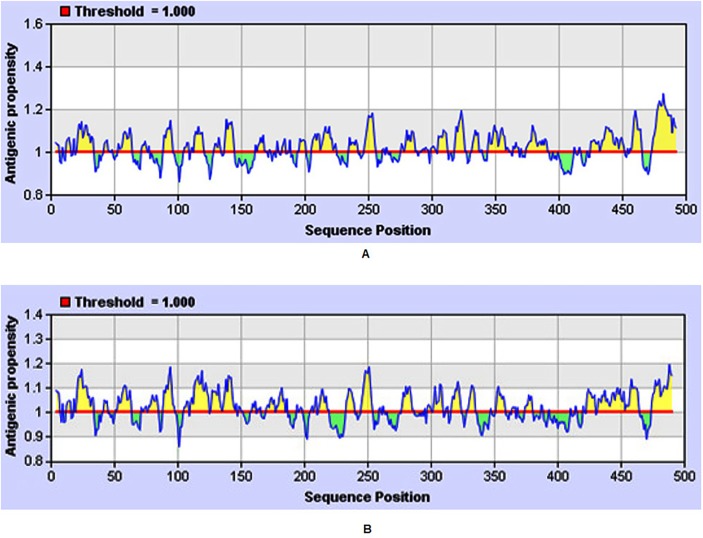
Graphical representation of predicted antigenicity of E protein. (A) DENV-2 Pakistani isolate. (B) DENV-3 Pakistani isolate.

The graphical representation of the predicted peptides of DENV-2 and DENV-3 on the basis of antigenic propensity (along y-axis) and sequence position (along x-axis) are shown in Fig. 1. The antigenic prospensity vary along the sequence length, the average antigenic prospensity value came out to be 1.026 with a minimum of 0.861 and a maximum of 1.272 for DENV-2 (Fig. 1A). Whereas, the antigenic prospensity of DENV-3 has the average value of 1.023 with a minimum of 0.861 and a maximum of 1.195 (Fig. 1B).

### Surface Accessibility and Flexibility

**Fig 2:**
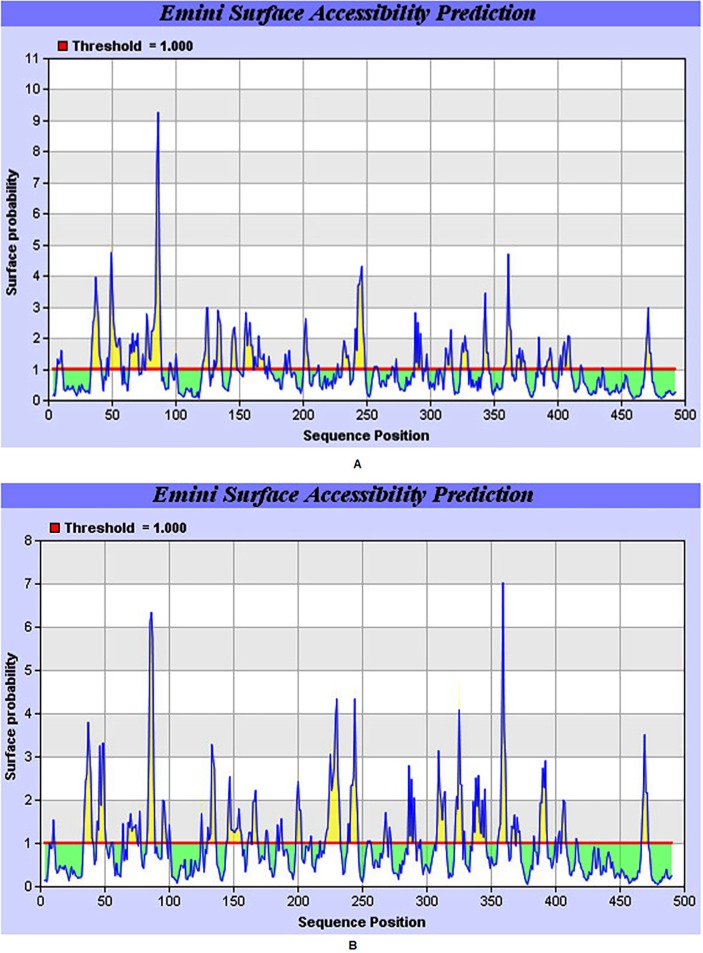
Graphical representation of surface accessibility prediction of E protein. (A) DENV-2 Pakistani isolate. (B) DENV-3 Pakistani isolate.

According to Emini et al, the surface probability of a hexapeptide greater than 1.0 (threshold) predicts that the sequence has increased probability to be found on the surface [25]. The graphical representation of the predicted peptides of DENV-2 and DENV-3 on the basis of surface probability (along y-axis) and sequence position (along x-axis) are shown in Fig. 2. The maximum surface probability value calculated by the software was 9.255 from amino acid position 84 to 89 for DENV-2. The sequence of the hexapeptide, according to the Emini Surfac Accessibility Prediction result data table (S1 Table) is 84EEQDKR89, where 86Q is the surface residue i.e., one with >20 Å distance to water. The minimum value of surface probability is 0.060 for peptides 457KILIGV460 and 458ILIGVV462 (at amino acid positions 457 to 460 and 458 to 463 respectively) as can be seen from Fig. 2A. On the other hand, the surface probability of DENV-3 has the a maximum value of 7.029. The sequence of the hexapeptide, according to the result table (S2 Table) is 357TKKEEP362, where the 359K is the surface residue. The minimum value of surface probability is 0.048 for peptides 477CIAIGI482 (Fig. 2B). The residues with the higher surface probabilty are vital candidates for the development of DENV peptide vaccine.

**Fig 3:**
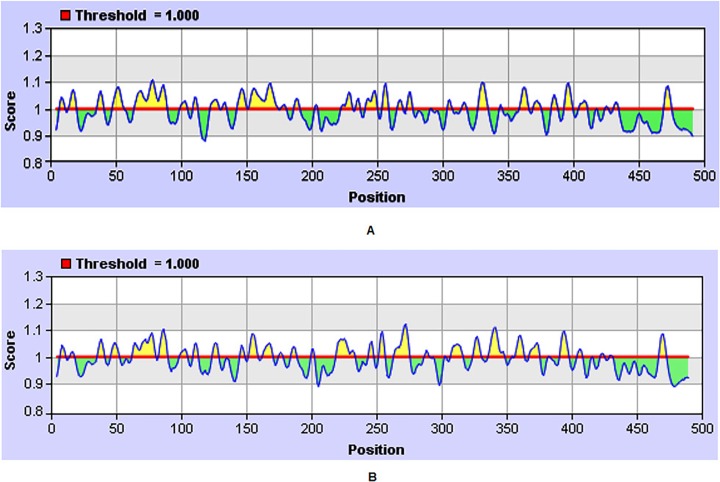
Graphical representation of flexibility prediction of E protein. (A) DENV-2 Pakistani isolate. (B) DENV-3 Pakistani isolate.

The Karplus and Schulz’s flexibility scale method calculates the B factor or temperature the factor that indicates vibrational motion of atoms within structure. Atoms in a well ordered structure has low B factor values,wheras the higher the B factor value, the more flexible structure it is [26]. The graphical forms of the results for DENV-2 and DENV-3 E protein surface flexibility are shown in Fig. 3 (A & B). For DENV-2 the maximum flexibilty score was 1.108 (hexapeptide: 75 to 81 a.a). The sequence of the heptapeptide, according to the result table (S3 Table) is 75PTQGEPS81, where 78G is the surface residue. The more ordered part of the structure has minimum score of 0.884(Fig. 3A). While, the flexbility of DENV-3 has the a maximum score of 1.122. According to the result table (S4 Table) the sequence of the heptapeptide, is 269QNSGGTS275, where the 272G is the surface residue. The minimum value that depicts the more ordered structural part is upto 0.892 (Fig. 3B). The predicted flexibility of E protein could be helpful in DENV vaccine development.

### Homology Modeling of DENV-2 and DENV-3 Envelope Proteins

**Fig 4:**
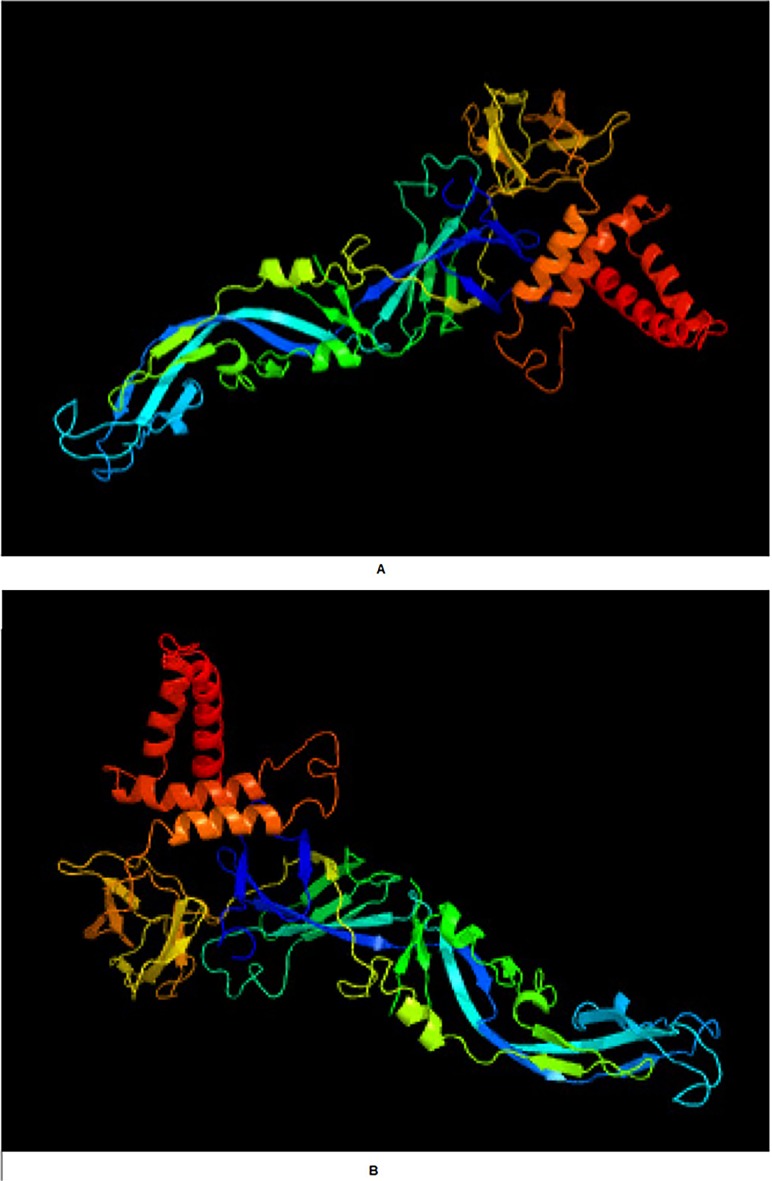
E protein structure model by using Phyre2 web server represented in cartoons. (A) DENV-2 Pakistani isolate. (B) DENV-3 Pakistani isolate.

Phyre2 web server found 69% maximum identity of DENV-2PK (495 a.a) E protein with Protein Data Bank (PDB): 4b03 chain A (electron cryomicroscopy structure of dengue virus serotype 1 envelope protein). The software used PDB: 4b03 chain A as a template and homology modeled the strucutre with 100% confidence (Fig. 4A). Whereas, the server established 77% identity of DENV-3PK (493 a.a) E protein with the same template and modeled the strucutre with 100% confidence and coverage (Fig. 4B). Confidence >90% shows that the core model is highly accurate with 2–4Å rmsd from native structure. While, percentage identity between sequence and template >30–40% indicate extremely high accuracy model.

### Structure-based Epitope Prediction

**Fig 5:**
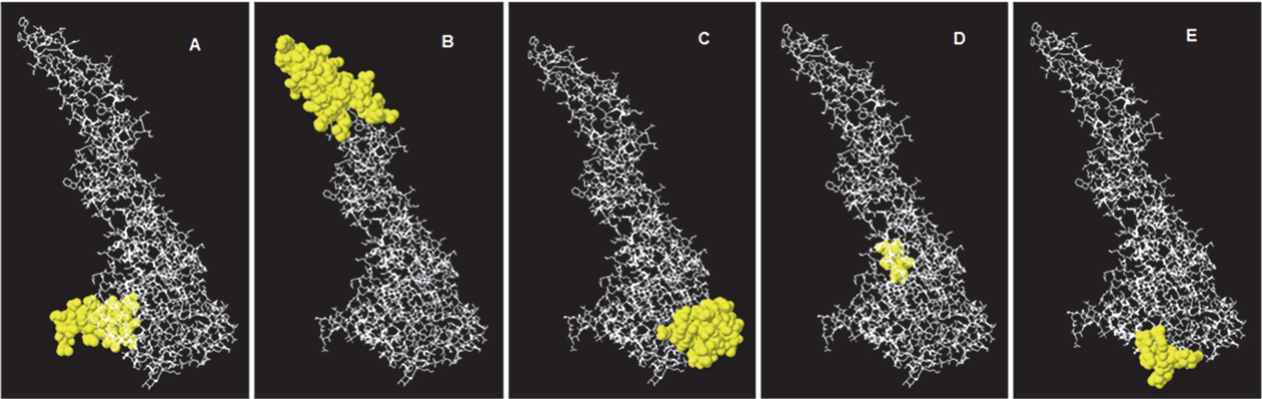
3D Representation of Discontinues Epitopes (A to E) of DENV-2 Pakistani isolates.

ElliPro is an advanced and accurate web tool for epitope prediction in 3D structures. This application set a co-relation between antigenicity, solvent accessibility and flexibility of a protein structure. Its efficient feature is to differentiate epitopes on the basis of protein-antibody interactions. For DENV-2 five discontinuous peptides having score value above 0.7 were selected. Score is also called Protrusion Index (PI) value which shows the percentage of protein atoms that extend beyond the molecular bulk (ellipsoid) and are involved in antibody binding. The highest probability of a discontinuous epitope was calculated as 90.3% (PI score: 0.903). Residues involved in discontinuous epitopes, their sequence location, number of residues and scores are given in Table 3 whereas, their positions on 3D structures are shown in Fig. 5 (A through E).

**Table 3:** Predicted discontinous antigenic epitopes of E protein of DENV-2 Pakistani isolate.

No.	Residues and their Positions	Number of Residues	Score	3D Structure
**1**	I457, L458, I459, G460, V461, V462, I463, T464, I466, G467, M468, N469, S470, R471, S472, T473, S474, L475, S476, V477, S478, L479, V480, L481, V482, G483, V484, T486, L487, Y488	30	0.903	Fig. 5A
**2**	T66, N67, T68, T69, T70, A71, S72, R73, C74, P75, T76, Q77, G78, E79, P80, S81, L82, N83, E84, E85, Q86, D87, K88, R89, F90, K93, H94, S95, M96, V97, D98, R99, G100, W101, G102, N103, G104, C105, G106, L107, F108, G109, K110, G111, G112, I113, V114, T115, C116, K241, N242, P243, H244, A245, K246, K247, Q248, D249, V250, V251	60	0.852	Fig. 5B
**3**	G296, M297, S298, Y299, S300, M301, C302, T303, G304, K305, F306, K307, V308, V309, K310, E311, R323, V324, Q325, Y326, E327, G328, D329, G330, S331, P332, C333, K334, I335, P336, I357, V358, T359, E360, K361, D362, S363, P364, V365, G381, V382, E383, P384, G385, Q386	45	0.783	Fig. 5C
**4**	A419, D421, F422, G423, S424, L425, G426, G427, V428	9	0.762	Fig. 5D
**5**	M340, D341, L342, E343, G374, D375, Y377, L387, K388, L389, S390, W391, F392, K393	14	0.712	Fig. 5E

DENV-3 has four discontinuous peptides having score value above 0.7. The highest probability of a discontinuous epitope was calculated as 90% (PI score: 0.900). Residues involved in discontinuous epitopes, their sequence location, number of residues and scores in Table 4.

**Table 4:** Predicted discontinous antigenic epitopes of E protein of DENV-3 Pakistani isolate.

No.	Residues and their Positions	Number of Residues	Score	3D Structure
**1**	I455, G456, I457, G458, V459, L460, L461, T462, I464, G465, L466, N467, S468, K469, N470, T471, S472, M473, S474, F475, S476, C477, I478, A479, I480, G481, I482, T484, L485, Y486	30	0.900	Fig. 6A
**2**	T66, N67, I68, T69, T70, D71, S72, R73, C74, P75, T76, Q77, G78, E79, A80, V81, L82, P83, E84, E85, Q86, D87, Q88, N89, Y90, K93, H94, T95, Y96, V97, D98, R99, G100, W101, G102, N103, G104, C105, G106, L107, F108, G109, K110, G111, S112, L113, V114, T115, C116, K239, N240, A241, H242, A243, K244, K245, Q246, E247	58	0.854	Fig. 6B
**3**	G294, M295, S296, Y297, A298, M299, C300, T301, N302, T303, F304, V305, L306, K307, K308, E309, K321, V322, E323, Y324, K325, G326, E327, D328, A329, P330, C331, K332, I333, P334, E338, D339, G340, Q341, G342, V355, V356, T357, K358, K359, E360, E361, P362, V363, G379, I380, G381, D382, N383, A384, L385, K386, I387, N388	54	0.774	Fig. 6C
**4**	A417, D419, F420, G421, S422, V423, G424, G425, V426, L427	10	0.753	Fig. 6D

**Fig 6:**
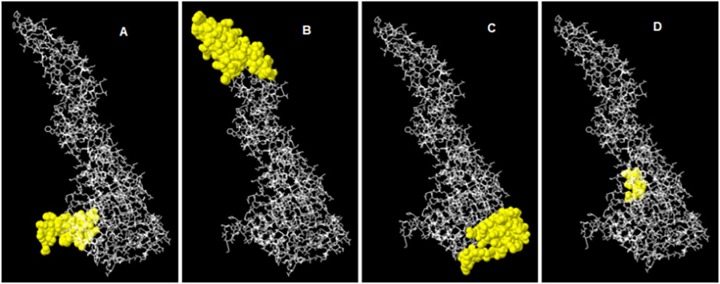
3D Representation of Discontinues Epitopes (A to D) of DENV-3 Pakistani isolates.

Their positions on globular structures are shown in Fig. 6 (A to D). The epitopes are represented by yellow surface and bulk of the E protein is represented in grey sticks. ElliPro has been tested on standard data of conformational antibody-protein complexes. In comparison to other available tools that require training, it is user friendly. The amino acid prospensities, residue solvent accessibility, spatial distribution and inter-molecular contacts are already integrated features in the tool.

### Conclusion

Prediction of B-cell epitopes has two main applications, diagnosis and vaccine development. Antibodies can be designed artificially to cross-react with the antigenic epitopes and used in viral infection diagnosis. Peptides representing antigenic determinants are attractive candidatesfor prophylactic and curative vaccines. In the present study, different tools were used to analyze various features of antigenic epitopes for DENV E protein which includes antigenecity, surface accessibility, flexibility, residue solvent accessibility, spatial distribution and inter-molecular contacts. Neutralizing monoclonal antibodies can be designed by using these linear and conformational epitopes against dengue virus that can work as an effective vaccine to save many precious lives. The epitopes predicted in this studyare important candidates which can be further used for preclinical and clinical testing in developing vaccines.

## Supporting Information

S1 Accession Number Dengue virus 2 isolate D2/Pakistan/2011-24/2011 envelope proteingene, partial cds. The reference sequence (Accession No. KF041224) was selected based on multiple sequence alignment results for DENV-2 Pakistani isolates and translated by EMBOSS-Transeq used in the present study.
(PDF)

S2 Accession Number Dengue virus 3 isolate D2/Pakistan/2011-24/2011 envelope protein gene, partial cds. The reference sequence (Accession No. KF041238) was selected based on multiple sequence alignment results for DENV-3 Pakistani isolates and translated by EMBOSS-Transeq was used in the present study.
(PDF)

S1 Table Emini Surface Accessibility Prediction Result Data for DENV2 Pakistani isolate. (PDF)

S2 Table Emini Surface Accessibility Prediction Result Data for DENV3 Pakistani isolate. (PDF)

S3 Table Karplus & Schulz Flexibility Prediction Result Data DENV2 Pakistani isolate. (PDF)

S4 Table Karplus & Schulz Flexibility Prediction Result Data DENV3 Pakistani isolate. (PDF)
