# Supplementary material for: Computational Prediction and Analysis of Envelop Glycoprotein Epitopes of DENV-2 and DENV-3 Pakistani Isolates: A First Step towards Dengue Vaccine Development
Source: PLoS One. 2015 Mar 16;10(3):e0119854. doi: 10.1371/journal.pone.0119854 (PMC4361635; doi:10.1371/journal.pone.0119854)
Supplement: S1 Accession Number — The reference sequence (Accession No. KF041224) was selected based on multiple sequence alignment results for DENV-2 Pakistani isolates and translated by EMBOSS-Transeq used in the present study. (PDF) [file pone.0119854.s001.pdf]

Nucleotide

Display Settings: GenBank

Dengue virus 2 isolate D2/Pakistan/2011-24/2011 envelope protein ge

GenBank: KF041224.1

FASTA Graphics PopSet

Go to:

```
LOCUS       KF041224                1485 bp    RNA     linear   VRL 23-DEC-2013
DEFINITION  Dengue virus 2 isolate D2/Pakistan/2011-24/2011 envelope protein
            gene, partial cds.
ACCESSION   KF041224
VERSION     KF041224.1  GI:567321261
KEYWORDS    .
SOURCE      Dengue virus 2
  ORGANISM  Dengue virus 2
            Viruses; ssRNA positive-strand viruses, no DNA stage; Flaviviridae;
            Flavivirus; Dengue virus group.
REFERENCE   1  (bases 1 to 1485)
  AUTHORS   Koo,C., Nasir,A., Hapuarachchi,H.C., Lee,K.S., Hasan,Z., Ng,L.C.
            and Khan,E.
  TITLE     Evolution and heterogeneity of multiple serotypes of Dengue virus
            in Pakistan, 2006-2011
  JOURNAL   Virol. J. 10, 275 (2013)
   PUBMED   24007412
  REMARK    Publication Status: Online-Only
REFERENCE   2  (bases 1 to 1485)
  AUTHORS   Koo,C., Nasir,A., Hapuarachchi,H.C., Lee,K.S., Hasan,Z. and Khan,E.
  TITLE     Direct Submission
  JOURNAL   Submitted (14-MAY-2013) Environmental Health Institute, National
            Environment Agency, 11, Biopolis Way, #06-05-08, Singapore 138667,
            Singapore
COMMENT     ##Assembly-Data-START##
            Assembly Method       :: Lasergene v. 8.0
            Sequencing Technology :: Sanger dideoxy sequencing
            ##Assembly-Data-END##
FEATURES             Location/Qualifiers
     source          1..1485
                     /organism="Dengue virus 2"
                     /mol_type="genomic RNA"
                     /serotype="2"
                     /isolate="D2/Pakistan/2011-24/2011"
                     /host="Homo sapiens"
                     /db_xref="taxon:11060"
                     /country="Pakistan"
                     /collection_date="2011"
     CDS             <1..>1485
                     /codon_start=1
                     /product="envelope protein"
                     /protein_id="AHC72396.1"
                     /db_xref="GI:567321262"
                     /translation="MRCIGISNRDFVEGVSGGSWVDIVLEHGSCVTTMAKNKPTLDFE
```

```
                     LIKTEAKQPATLRKYCIEAKLTNTTTASRCPTQGEPSLNEEQDKRFVCKHSMVDRGWG
                     NGCGLFGKGGIVTCAMFTCKKNMEGKIVQPENLEYTIVVTPHSGEENAVGNDTGKHGK
                     EIKVTPQSSITEAELTGYGTVTMECSPRTGLDFNEMVLLQMENKAWLVHRQWFLDLPL
                     PWLPGADIQGSNWIQKETLVTFKNPHAKKQDVVVLGSQEGAMHTALTGATEIQMSSGN
                     LLFTGHLKCRLRMDKLQLKGMSYSMCTGKFKVVKEIAETQHGTIVVRVQYEGDGSPCK
                     IPFEIMDLEKRHVLGRLITVNPIVTEKDSPVNIEAEPPFGDSYIIIGVEPGQLKLSWF
                     KKGSSIGQMFETTMRGAKRMAILGDTAWDFGSLGGVFTSIGKALHQVFGAIYGAAFSG
                     VSWTMKILIGVVITWIGMNSRSTSLSVSLVLVGVVTLYLGVMVQA"
ORIGIN
        1 atgcgttgta ttggaatatc aaatagagac tttgtggaag gggtttcagg aggaagctgg
       61 gttgacatag tcttagaaca tggaagctgt gtgacaacga tggcaaaaaa caaaccaaca
      121 ttggactttg aactgataaa aacagaagcc aagcaacctg ccactctaag gaagtactgt
      181 atagaggcaa agctgaccaa cacaacaaca gcatctcgat gcccaacaca aggggaaccc
      241 agcctaaatg aagaacagga caaaaggttt gtctgcaaac attccatggt agacagagga
      301 tggggaaatg gatgtggatt atttggaaag ggaggcattg tgacctgtgc tatgttcaca
      361 tgcaaaaaaa acatggaagg gaaaattgtg caaccagaaa acctggaata caccattgtg
      421 gtaacacctc attcagggga agagaatgca gttggaaatg acacaggaaa acatggcaag
      481 gaaatcaaag tgacaccaca gagttccatt acagaagcag aactgacagg ctacggcact
      541 gtcacgatgg agtgctctcc aagaacgggc ctcgacttca atgagatggt gttgctgcaa
      601 atggaaaaca aggcttggct ggtgcacagg caatggttct tagacctgcc gttaccatgg
      661 ctgcccggag cggacataca aggatcaaat tggatacaga aggagacgtt ggtcactttc
      721 aaaaaccccc atgcgaagaa acaggatgtt gttgttttag gatcccaaga aggggctatg
      781 catacagcac tcacaggggc cacggaaatc cagatgtcat caggaaacct actgttcaca
      841 ggacatctca agtgcaggct gagaatggat aaattacagc tcaaaggaat gtcatactct
      901 atgtgtacag gaaagtttaa agttgtaaag gaaatagcag aaacacaaca tggaacaata
      961 gttgtcagag tacaatatga aggggatggc tctccatgta aaatcccttt tgagataatg
     1021 gatttggaaa aaagacatgt cttaggtcgc ctgattacag tcaacccaat cgtcacagaa
     1081 aaagacagtc cagtcaacat agaagcagaa cccccattcg gagacagcta catcattata
     1141 ggagtagaac cgggacaact gaagctcagc tggtttaaga aaggaagctc aatcggccaa
     1201 atgttcgaaa caacaatgag aggagcgaag agaatggcca ttttaggtga cacagcctgg
     1261 gattttggat ccctgggagg agtgttcaca tctataggaa aggccctcca ccaagttttt
     1321 ggagctatct atggggctgc cttcagtggg gtttcatgga ctatgaaaat cctcatagga
     1381 gttgtcatca catggatagg aatgaattca cgtagcacct cactgtctgt gtcactagta
     1441 ttagtgggag tcgtgacatt gtacttggga gttatggtgc aggct
//
```
