# Supplementary material for: Computational Prediction and Analysis of Envelop Glycoprotein Epitopes of DENV-2 and DENV-3 Pakistani Isolates: A First Step towards Dengue Vaccine Development
Source: PLoS One. 2015 Mar 16;10(3):e0119854. doi: 10.1371/journal.pone.0119854 (PMC4361635; doi:10.1371/journal.pone.0119854)
Supplement: S2 Accession Number — The reference sequence (Accession No. KF041238) was selected based on multiple sequence alignment results for DENV-3 Pakistani isolates and translated by EMBOSS-Transeq was used in the present study. (PDF) [file pone.0119854.s002.pdf]

Nucleotide

**Display Settings:** GenBank

# Dengue virus 3 isolate D3/Pakistan/56139/2006 envelope protein gene

GenBank: KF041238.1

FASTA Graphics PopSet

Go to:

```
LOCUS       KF041238                1479 bp    RNA     linear   VRL 23-DEC-2013
DEFINITION  Dengue virus 3 isolate D3/Pakistan/56139/2006 envelope protein
            gene, partial cds.
ACCESSION   KF041238
VERSION     KF041238.1  GI:567319903
KEYWORDS    .
SOURCE      Dengue virus 3
  ORGANISM  Dengue virus 3
            Viruses; ssRNA positive-strand viruses, no DNA stage; Flaviviridae;
            Flavivirus; Dengue virus group.
REFERENCE   1  (bases 1 to 1479)
  AUTHORS   Koo,C., Nasir,A., Hapuarachchi,H.C., Lee,K.S., Hasan,Z., Ng,L.C.
            and Khan,E.
  TITLE     Evolution and heterogeneity of multiple serotypes of Dengue virus
            in Pakistan, 2006-2011
  JOURNAL   Virol. J. 10, 275 (2013)
   PUBMED   24007412
  REMARK    Publication Status: Online-Only
REFERENCE   2  (bases 1 to 1479)
  AUTHORS   Koo,C., Nasir,A., Hapuarachchi,H.C., Lee,K.S., Hasan,Z. and Khan,E.
  TITLE     Direct Submission
  JOURNAL   Submitted (14-MAY-2013) Environmental Health Institute, National
            Environment Agency, 11, Biopolis Way, #06-05-08, Singapore 138667,
            Singapore
COMMENT     ##Assembly-Data-START##
            Assembly Method       :: Lasergene v. 8.0
            Sequencing Technology :: Sanger dideoxy sequencing
            ##Assembly-Data-END##
FEATURES             Location/Qualifiers
     source          1..1479
                     /organism="Dengue virus 3"
                     /mol_type="genomic RNA"
                     /serotype="3"
                     /isolate="D3/Pakistan/56139/2006"
                     /host="Homo sapiens"
                     /db_xref="taxon:11069"
                     /country="Pakistan: Karachi"
                     /collection_date="2006"
                     /note="genotype: III"
     CDS             <1..>1479
                     /codon_start=1
                     /product="envelope protein"
                     /protein_id="AHC72410.1"
                     /db_xref="GI:567319904"
```
