# Supplementary material for: Computational Prediction and Analysis of Envelop Glycoprotein Epitopes of DENV-2 and DENV-3 Pakistani Isolates: A First Step towards Dengue Vaccine Development
Source: PLoS One. 2015 Mar 16;10(3):e0119854. doi: 10.1371/journal.pone.0119854 (PMC4361635; doi:10.1371/journal.pone.0119854)
Supplement: S1 Table — (PDF) [file pone.0119854.s003.pdf]

# IEDB Analysis Resource

Antibody Epitope Prediction | Example Sequences | Tutorial | External Links | Reference | Download | Contact

## Emini Surface Accessibility Prediction Result Data Table

**Average:** 1.000 **Minimum:** 0.060 **Maximum:** 9.255 Download data to file

| Position ▲ ▼ | Residue | Peptide start position | Peptide end position | Peptide | Score ▲ ▼ |
|---|---|---|---|---|---|
| 3 | **C** | 1 | 6 | MR**C**IGI | 0.138 |
| 4 | **I** | 2 | 7 | RC**I**GIS | 0.186 |
| 5 | **G** | 3 | 8 | CI**G**ISN | 0.153 |
| 6 | **I** | 4 | 9 | IG**I**SNR | 0.559 |
| 7 | **S** | 5 | 10 | GI**S**NRD | 1.332 |
| 8 | **N** | 6 | 11 | IS**N**RDF | 1.165 |
| 9 | **R** | 7 | 12 | SN**R**DFV | 1.234 |
| 10 | **D** | 8 | 13 | NR**D**FVE | 1.595 |
| 11 | **F** | 9 | 14 | RD**F**VEG | 0.981 |
| 12 | **V** | 10 | 15 | DF**V**EGV | 0.372 |
| 13 | **E** | 11 | 16 | FV**E**GVS | 0.298 |
| 14 | **G** | 12 | 17 | VE**G**VSG | 0.341 |
| 15 | **V** | 13 | 18 | EG**V**SGG | 0.455 |
| 16 | **S** | 14 | 19 | GV**S**GGS | 0.352 |
| 17 | **G** | 15 | 20 | VS**G**GSW | 0.374 |
| 18 | **G** | 16 | 21 | SG**G**SWV | 0.374 |
| 19 | **S** | 17 | 22 | GG**S**WVD | 0.466 |
| 20 | **W** | 18 | 23 | GS**W**VDI | 0.330 |
| 21 | **V** | 19 | 24 | SW**V**DIV | 0.248 |
| 22 | **D** | 20 | 25 | WV**D**IVL | 0.152 |
| 23 | **I** | 21 | 26 | VD**I**VLE | 0.251 |
| 24 | **V** | 22 | 27 | DI**V**LEH | 0.460 |

| | | | | | |
|---|---|---|---|---|---|
| 25 | **L** | 23 | 28 | IV**L**EHG | 0.273 |
| 26 | **E** | 24 | 29 | VL**E**HGS | 0.521 |
| 27 | **H** | 25 | 30 | LE**H**GSC | 0.376 |
| 28 | **G** | 26 | 31 | EH**G**SCV | 0.339 |
| 29 | **S** | 27 | 32 | HG**S**CVT | 0.282 |
| 30 | **C** | 28 | 33 | GS**C**VTT | 0.299 |
| 31 | **V** | 29 | 34 | SC**V**TTM | 0.299 |
| 32 | **T** | 30 | 35 | CV**T**TMA | 0.226 |
| 33 | **T** | 31 | 36 | VT**T**MAK | 0.842 |
| 34 | **M** | 32 | 37 | TT**M**AKN | 1.824 |
| 35 | **A** | 33 | 38 | TM**A**KNK | 2.528 |
| 36 | **K** | 34 | 39 | MA**K**NKP | 2.708 |
| 37 | **N** | 35 | 40 | AK**N**KPT | 3.950 |
| 38 | **K** | 36 | 41 | KN**K**PTL | 3.224 |
| 39 | **P** | 37 | 42 | NK**P**TLD | 2.692 |
| 40 | **T** | 38 | 43 | KP**T**LDF | 1.450 |
| 41 | **L** | 39 | 44 | PT**L**DFE | 1.255 |
| 42 | **D** | 40 | 45 | TL**D**FEL | 0.670 |
| 43 | **F** | 41 | 46 | LD**F**ELI | 0.325 |
| 44 | **E** | 42 | 47 | DF**E**LIK | 0.789 |
| 45 | **L** | 43 | 48 | FE**L**IKT | 0.682 |
| 46 | **I** | 44 | 49 | EL**I**KTE | 1.363 |
| 47 | **K** | 45 | 50 | LI**K**TEA | 0.795 |
| 48 | **T** | 46 | 51 | IK**T**EAK | 1.928 |
| 49 | **E** | 47 | 52 | KT**E**AKQ | 4.764 |
| 50 | **A** | 48 | 53 | TE**A**KQP | 3.683 |
| 51 | **K** | 49 | 54 | EA**K**QPA | 2.578 |
| 52 | **Q** | 50 | 55 | AK**Q**PAT | 2.149 |
| 53 | **P** | 51 | 56 | KQ**P**ATL | 1.754 |
| 54 | **A** | 52 | 57 | QP**A**TLR | 1.718 |
| 55 | **T** | 53 | 58 | PA**T**LRK | 1.984 |
| 56 | **L** | 54 | 59 | AT**L**RKY | 2.010 |
| 57 | **R** | 55 | 60 | TL**R**KYC | 1.067 |
| 58 | **K** | 56 | 61 | LR**K**YCI | 0.518 |
| 59 | **Y** | 57 | 62 | RK**Y**CIE | 1.088 |
| 60 | **C** | 58 | 63 | KY**C**IEA | 0.561 |

| | | | | | |
|---|---|---|---|---|---|
| 61 | **I** | 59 | 64 | YC**I**EAK | 0.561 |
| 62 | **E** | 60 | 65 | CI**E**AKL | 0.295 |
| 63 | **A** | 61 | 66 | IE**A**KLT | 0.795 |
| 64 | **K** | 62 | 67 | EA**K**LTN | 1.824 |
| 65 | **L** | 63 | 68 | AK**L**TNT | 1.520 |
| 66 | **T** | 64 | 69 | KL**T**NTT | 2.172 |
| 67 | **N** | 65 | 70 | LT**N**TTT | 1.567 |
| 68 | **T** | 66 | 71 | TN**T**TTA | 1.920 |
| 69 | **T** | 67 | 72 | NT**T**TAS | 1.783 |
| 70 | **T** | 68 | 73 | TT**T**ASR | 2.171 |
| 71 | **A** | 69 | 74 | TT**A**SRC | 0.806 |
| 72 | **S** | 70 | 75 | TA**S**RCP | 0.864 |
| 73 | **R** | 71 | 76 | AS**R**CPT | 0.864 |
| 74 | **C** | 72 | 77 | SR**C**PTQ | 1.481 |
| 75 | **P** | 73 | 78 | RC**P**TQG | 1.094 |
| 76 | **T** | 74 | 79 | CP**T**QGE | 0.967 |
| 77 | **Q** | 75 | 80 | PT**Q**GEP | 2.790 |
| 78 | **G** | 76 | 81 | TQ**G**EPS | 2.418 |
| 79 | **E** | 77 | 82 | QG**E**PSL | 1.382 |
| 80 | **P** | 78 | 83 | GE**P**SLN | 1.283 |
| 81 | **S** | 79 | 84 | EP**S**LNE | 2.245 |
| 82 | **L** | 80 | 85 | PS**L**NEE | 2.245 |
| 83 | **N** | 81 | 86 | SL**N**EEQ | 2.515 |
| 84 | **E** | 82 | 87 | LN**E**EQD | 3.134 |
| 85 | **E** | 83 | 88 | NE**E**QDK | 7.599 |
| 86 | **Q** | 84 | 89 | EE**Q**DKR | 9.255 (maximum) |
| 87 | **D** | 85 | 90 | EQ**D**KRF | 4.627 |
| 88 | **K** | 86 | 91 | QD**K**RFV | 1.983 |
| 89 | **R** | 87 | 92 | DK**R**FVC | 0.614 |
| 90 | **F** | 88 | 93 | KR**F**VCK | 0.735 |
| 91 | **V** | 89 | 94 | RF**V**CKH | 0.500 |
| 92 | **C** | 90 | 95 | FV**C**KHS | 0.342 |
| 93 | **K** | 91 | 96 | VC**K**HSM | 0.391 |
| 94 | **H** | 92 | 97 | CK**H**SMV | 0.391 |
| 95 | **S** | 93 | 98 | KH**S**MVD | 1.218 |
| 96 | **M** | 94 | 99 | HS**M**VDR | 1.193 |

| | | | | | |
|---|---|---|---|---|---|
| 97 | **V** | 95 | 100 | SM**V**DRG | 0.868 |
| 98 | **D** | 96 | 101 | MV**D**RGW | 0.681 |
| 99 | **R** | 97 | 102 | VD**R**GWG | 0.681 |
| 100 | **G** | 98 | 103 | DR**G**WGN | 1.475 |
| 101 | **W** | 99 | 104 | RG**W**GNG | 0.874 |
| 102 | **G** | 100 | 105 | GW**G**NGC | 0.239 |
| 103 | **N** | 101 | 106 | WG**N**GCG | 0.239 |
| 104 | **G** | 102 | 107 | GN**G**CGL | 0.188 |
| 105 | **C** | 103 | 108 | NG**C**GLF | 0.164 |
| 106 | **G** | 104 | 109 | GC**G**LFG | 0.101 |
| 107 | **L** | 105 | 110 | CG**L**FGK | 0.204 |
| 108 | **F** | 106 | 111 | GL**F**GKG | 0.377 |
| 109 | **G** | 107 | 112 | LF**G**KGG | 0.377 |
| 110 | **K** | 108 | 113 | FG**K**GGI | 0.320 |
| 111 | **G** | 109 | 114 | GK**G**GIV | 0.275 |
| 112 | **G** | 110 | 115 | KG**G**IVT | 0.401 |
| 113 | **I** | 111 | 116 | GG**I**VTC | 0.107 |
| 114 | **V** | 112 | 117 | GI**V**TCA | 0.110 |
| 115 | **T** | 113 | 118 | IV**T**CAM | 0.110 |
| 116 | **C** | 114 | 119 | VT**C**AMF | 0.135 |
| 117 | **A** | 115 | 120 | TC**A**MFT | 0.263 |
| 118 | **M** | 116 | 121 | CA**M**FTC | 0.098 |
| 119 | **F** | 117 | 122 | AM**F**TCK | 0.365 |
| 120 | **T** | 118 | 123 | MF**T**CKK | 0.722 |
| 121 | **C** | 119 | 124 | FT**C**KKN | 1.174 |
| 122 | **K** | 120 | 125 | TC**K**KNM | 1.341 |
| 123 | **K** | 121 | 126 | CK**K**NME | 1.609 |
| 124 | **N** | 122 | 127 | KK**N**MEG | 2.971 |
| 125 | **M** | 123 | 128 | KN**M**EGK | 2.971 |
| 126 | **E** | 124 | 129 | NM**E**GKI | 1.042 |
| 127 | **G** | 125 | 130 | ME**G**KIV | 0.481 |
| 128 | **K** | 126 | 131 | EG**K**IVQ | 0.841 |
| 129 | **I** | 127 | 132 | GK**I**VQP | 0.751 |
| 130 | **V** | 128 | 133 | KI**V**QPE | 1.314 |
| 131 | **Q** | 129 | 134 | IV**Q**PEN | 1.057 |
| 132 | **P** | 130 | 135 | VQ**P**ENL | 1.243 |

| | | | | | |
|---|---|---|---|---|---|
| 133 | **E** | 131 | 136 | QP**E**NLE | 2.901 |
| 134 | **N** | 132 | 137 | PE**N**LEY | 2.625 |
| 135 | **L** | 133 | 138 | EN**L**EYT | 2.450 |
| 136 | **E** | 134 | 139 | NL**E**YTI | 0.992 |
| 137 | **Y** | 135 | 140 | LE**Y**TIV | 0.458 |
| 138 | **T** | 136 | 141 | EY**T**IVV | 0.412 |
| 139 | **I** | 137 | 142 | YT**I**VVT | 0.343 |
| 140 | **V** | 138 | 143 | TI**V**VTP | 0.339 |
| 141 | **V** | 139 | 144 | IV**V**TPH | 0.319 |
| 142 | **T** | 140 | 145 | VV**T**PHS | 0.611 |
| 143 | **P** | 141 | 146 | VT**P**HSG | 0.814 |
| 144 | **H** | 142 | 147 | TP**H**SGE | 1.900 |
| 145 | **S** | 143 | 148 | PH**S**GEE | 2.280 |
| 146 | **G** | 144 | 149 | HS**G**EEN | 2.371 |
| 147 | **E** | 145 | 150 | SG**E**ENA | 1.760 |
| 148 | **E** | 146 | 151 | GE**E**NAV | 0.975 |
| 149 | **N** | 147 | 152 | EE**N**AVG | 0.975 |
| 150 | **A** | 148 | 153 | EN**A**VGN | 0.905 |
| 151 | **V** | 149 | 154 | NA**V**GND | 0.873 |
| 152 | **G** | 150 | 155 | AV**G**NDT | 0.783 |
| 153 | **N** | 151 | 156 | VG**N**DTG | 0.767 |
| 154 | **D** | 152 | 157 | GN**D**TGK | 2.068 |
| 155 | **T** | 153 | 158 | ND**T**GKH | 2.843 |
| 156 | **G** | 154 | 159 | DT**G**KHG | 1.750 |
| 157 | **K** | 155 | 160 | TG**K**HGK | 2.095 |
| 158 | **H** | 156 | 161 | GK**H**GKE | 2.514 |
| 159 | **G** | 157 | 162 | KH**G**KEI | 1.781 |
| 160 | **K** | 158 | 163 | HG**K**EIK | 1.781 |
| 161 | **E** | 159 | 164 | GK**E**IKV | 0.971 |
| 162 | **I** | 160 | 165 | KE**I**KVT | 1.417 |
| 163 | **K** | 161 | 166 | EI**K**VTP | 1.095 |
| 164 | **V** | 162 | 167 | IK**V**TPQ | 1.095 |
| 165 | **T** | 163 | 168 | KV**T**PQS | 2.094 |
| 166 | **P** | 164 | 169 | VT**P**QSS | 1.403 |
| 167 | **Q** | 165 | 170 | TP**Q**SSI | 1.325 |
| 168 | **S** | 166 | 171 | PQ**S**SIT | 1.325 |

| | | | | | |
|---|---|---|---|---|---|
| 169 | **S** | 167 | 172 | QS**S**ITE | 1.484 |
| 170 | **I** | 168 | 173 | SS**I**TEA | 0.866 |
| 171 | **T** | 169 | 174 | SI**T**EAE | 1.119 |
| 172 | **E** | 170 | 175 | IT**E**AEL | 0.689 |
| 173 | **A** | 171 | 176 | TE**A**ELT | 1.418 |
| 174 | **E** | 172 | 177 | EA**E**LTG | 0.972 |
| 175 | **L** | 173 | 178 | AE**L**TGY | 0.880 |
| 176 | **T** | 174 | 179 | EL**T**GYG | 0.862 |
| 177 | **G** | 175 | 180 | LT**G**YGT | 0.718 |
| 178 | **Y** | 176 | 181 | TG**Y**GTV | 0.646 |
| 179 | **G** | 177 | 182 | GY**G**TVT | 0.646 |
| 180 | **T** | 178 | 183 | YG**T**VTM | 0.646 |
| 181 | **V** | 179 | 184 | GT**V**TME | 0.714 |
| 182 | **T** | 180 | 185 | TV**T**MEC | 0.387 |
| 183 | **M** | 181 | 186 | VT**M**ECS | 0.359 |
| 184 | **E** | 182 | 187 | TM**E**CSP | 0.748 |
| 185 | **C** | 183 | 188 | ME**C**SPR | 1.016 |
| 186 | **S** | 184 | 189 | EC**S**PRT | 1.481 |
| 187 | **P** | 185 | 190 | CS**P**RTG | 0.846 |
| 188 | **R** | 186 | 191 | SP**R**TGL | 1.302 |
| 189 | **T** | 187 | 192 | PR**T**GLD | 1.623 |
| 190 | **G** | 188 | 193 | RT**G**LDF | 0.909 |
| 191 | **L** | 189 | 194 | TG**L**DFN | 0.746 |
| 192 | **D** | 190 | 195 | GL**D**FNE | 0.895 |
| 193 | **F** | 191 | 196 | LD**F**NEM | 0.895 |
| 194 | **N** | 192 | 197 | DF**N**EMV | 0.806 |
| 195 | **E** | 193 | 198 | FN**E**MVL | 0.398 |
| 196 | **M** | 194 | 199 | NE**M**VLL | 0.379 |
| 197 | **V** | 195 | 200 | EM**V**LLQ | 0.408 |
| 198 | **L** | 196 | 201 | MV**L**LQM | 0.233 |
| 199 | **L** | 197 | 202 | VL**L**QME | 0.408 |
| 200 | **Q** | 198 | 203 | LL**Q**MEN | 0.884 |
| 201 | **M** | 199 | 204 | LQ**M**ENK | 2.144 |
| 202 | **E** | 200 | 205 | QM**E**NKA | 2.627 |
| 203 | **N** | 201 | 206 | ME**N**KAW | 1.595 |
| 204 | **K** | 202 | 207 | EN**K**AWL | 1.329 |

| | | | | | |
|---|---|---|---|---|---|
| 205 | **A** | 203 | 208 | NK**A**WLV | 0.570 |
| 206 | **W** | 204 | 209 | KA**W**LVH | 0.482 |
| 207 | **L** | 205 | 210 | AW**L**VHR | 0.472 |
| 208 | **V** | 206 | 211 | WL**V**HRQ | 0.809 |
| 209 | **H** | 207 | 212 | LV**H**RQW | 0.809 |
| 210 | **R** | 208 | 213 | VH**R**QWF | 0.850 |
| 211 | **Q** | 209 | 214 | HR**Q**WFL | 0.944 |
| 212 | **W** | 210 | 215 | RQ**W**FLD | 1.159 |
| 213 | **F** | 211 | 216 | QW**F**LDL | 0.488 |
| 214 | **L** | 212 | 217 | WF**L**DLP | 0.436 |
| 215 | **D** | 213 | 218 | FL**D**LPL | 0.342 |
| 216 | **L** | 214 | 219 | LD**L**PLP | 0.610 |
| 217 | **P** | 215 | 220 | DL**P**LPW | 0.778 |
| 218 | **L** | 216 | 221 | LP**L**PWL | 0.384 |
| 219 | **P** | 217 | 222 | PL**P**WLP | 0.720 |
| 220 | **W** | 218 | 223 | LP**W**LPG | 0.461 |
| 221 | **L** | 219 | 224 | PW**L**PGA | 0.565 |
| 222 | **P** | 220 | 225 | WL**P**GAD | 0.610 |
| 223 | **G** | 221 | 226 | LP**G**ADI | 0.407 |
| 224 | **A** | 222 | 227 | PG**A**DIQ | 0.854 |
| 225 | **D** | 223 | 228 | GA**D**IQG | 0.546 |
| 226 | **I** | 224 | 229 | AD**I**QGS | 0.740 |
| 227 | **Q** | 225 | 230 | DI**Q**GSN | 1.178 |
| 228 | **G** | 226 | 231 | IQ**G**SNW | 0.742 |
| 229 | **S** | 227 | 232 | QG**S**NWI | 0.742 |
| 230 | **N** | 228 | 233 | GS**N**WIQ | 0.742 |
| 231 | **W** | 229 | 234 | SN**W**IQK | 1.499 |
| 232 | **I** | 230 | 235 | NW**I**QKE | 1.937 |
| 233 | **Q** | 231 | 236 | WI**Q**KET | 1.738 |
| 234 | **K** | 232 | 237 | IQ**K**ETL | 1.363 |
| 235 | **E** | 233 | 238 | QK**E**TLV | 1.443 |
| 236 | **T** | 234 | 239 | KE**T**LVT | 1.203 |
| 237 | **L** | 235 | 240 | ET**L**VTF | 0.521 |
| 238 | **V** | 236 | 241 | TL**V**TFK | 0.601 |
| 239 | **T** | 237 | 242 | LV**T**FKN | 0.670 |
| 240 | **F** | 238 | 243 | VT**F**KNP | 1.256 |

| | | | | | |
|---|---|---|---|---|---|
| 241 | **K** | 239 | 244 | TF**K**NPH | 2.303 |
| 242 | **N** | 240 | 245 | FK**N**PHA | 1.612 |
| 243 | **P** | 241 | 246 | KN**P**HAK | 3.724 |
| 244 | **H** | 242 | 247 | NP**H**AKK | 3.724 |
| 245 | **A** | 243 | 248 | PH**A**KKQ | 4.010 |
| 246 | **K** | 244 | 249 | HA**K**KQD | 4.331 |
| 247 | **K** | 245 | 250 | AK**K**QDV | 2.362 |
| 248 | **Q** | 246 | 251 | KK**Q**DVV | 1.736 |
| 249 | **D** | 247 | 252 | KQ**D**VVV | 0.644 |
| 250 | **V** | 248 | 253 | QD**V**VVL | 0.266 |
| 251 | **V** | 249 | 254 | DV**V**VLG | 0.152 |
| 252 | **V** | 250 | 255 | VV**V**LGS | 0.122 |
| 253 | **L** | 251 | 256 | VV**L**GSQ | 0.284 |
| 254 | **G** | 252 | 257 | VL**G**SQE | 0.663 |
| 255 | **S** | 253 | 258 | LG**S**QEG | 0.884 |
| 256 | **Q** | 254 | 259 | GS**Q**EGA | 1.083 |
| 257 | **E** | 255 | 260 | SQ**E**GAM | 1.083 |
| 258 | **G** | 256 | 261 | QE**G**AMH | 1.100 |
| 259 | **A** | 257 | 262 | EG**A**MHT | 0.917 |
| 260 | **M** | 258 | 263 | GA**M**HTA | 0.535 |
| 261 | **H** | 259 | 264 | AM**H**TAL | 0.446 |
| 262 | **T** | 260 | 265 | MH**T**ALT | 0.636 |
| 263 | **A** | 261 | 266 | HT**A**LTG | 0.636 |
| 264 | **L** | 262 | 267 | TA**L**TGA | 0.473 |
| 265 | **T** | 263 | 268 | AL**T**GAT | 0.473 |
| 266 | **G** | 264 | 269 | LT**G**ATE | 0.810 |
| 267 | **A** | 265 | 270 | TG**A**TEI | 0.689 |
| 268 | **T** | 266 | 271 | GA**T**EIQ | 0.826 |
| 269 | **E** | 267 | 272 | AT**E**IQM | 0.826 |
| 270 | **I** | 268 | 273 | TE**I**QMS | 1.096 |
| 271 | **Q** | 269 | 274 | EI**Q**MSS | 1.018 |
| 272 | **M** | 270 | 275 | IQ**M**SSG | 0.582 |
| 273 | **S** | 271 | 276 | QM**S**SGN | 1.334 |
| 274 | **S** | 272 | 277 | MS**S**GNL | 0.635 |
| 275 | **G** | 273 | 278 | SS**G**NLL | 0.529 |
| 276 | **N** | 274 | 279 | SG**N**LLF | 0.342 |

| | | | | | |
|---|---|---|---|---|---|
| 277 | **L** | 275 | 280 | GN**L**LFT | 0.368 |
| 278 | **L** | 276 | 281 | NL**L**FTG | 0.368 |
| 279 | **F** | 277 | 282 | LL**F**TGH | 0.312 |
| 280 | **T** | 278 | 283 | LF**T**GHL | 0.312 |
| 281 | **G** | 279 | 284 | FT**G**HLK | 0.756 |
| 282 | **H** | 280 | 285 | TG**H**LKC | 0.468 |
| 283 | **L** | 281 | 286 | GH**L**KCR | 0.635 |
| 284 | **K** | 282 | 287 | HL**K**CRL | 0.529 |
| 285 | **C** | 283 | 288 | LK**C**RLR | 0.762 |
| 286 | **R** | 284 | 289 | KC**R**LRM | 0.914 |
| 287 | **L** | 285 | 290 | CR**L**RMD | 0.763 |
| 288 | **R** | 286 | 291 | RL**R**MDK | 2.848 |
| 289 | **M** | 287 | 292 | LR**M**DKL | 1.199 |
| 290 | **D** | 288 | 293 | RM**D**KLQ | 2.518 |
| 291 | **K** | 289 | 294 | MD**K**LQL | 1.060 |
| 292 | **L** | 290 | 295 | DK**L**QLK | 2.143 |
| 293 | **Q** | 291 | 296 | KL**Q**LKG | 1.270 |
| 294 | **L** | 292 | 297 | LQ**L**KGM | 0.628 |
| 295 | **K** | 293 | 298 | QL**K**GMS | 1.021 |
| 296 | **G** | 294 | 299 | LK**G**MSY | 0.924 |
| 297 | **M** | 295 | 300 | KG**M**SYS | 1.501 |
| 298 | **S** | 296 | 301 | GM**S**YSM | 0.743 |
| 299 | **Y** | 297 | 302 | MS**Y**SMC | 0.402 |
| 300 | **S** | 298 | 303 | SY**S**MCT | 0.587 |
| 301 | **M** | 299 | 304 | YS**M**CTG | 0.433 |
| 302 | **C** | 300 | 305 | SM**C**TGK | 0.553 |
| 303 | **T** | 301 | 306 | MC**T**GKF | 0.357 |
| 304 | **G** | 302 | 307 | CT**G**KFK | 0.722 |
| 305 | **K** | 303 | 308 | TG**K**FKV | 1.000 |
| 306 | **F** | 304 | 309 | GK**F**KVV | 0.514 |
| 307 | **K** | 305 | 310 | KF**K**VVK | 1.039 |
| 308 | **V** | 306 | 311 | FK**V**VKE | 0.900 |
| 309 | **V** | 307 | 312 | KV**V**KEI | 0.729 |
| 310 | **K** | 308 | 313 | VV**K**EIA | 0.368 |
| 311 | **E** | 309 | 314 | VK**E**IAE | 0.859 |
| 312 | **I** | 310 | 315 | KE**I**AET | 1.670 |

| | | | | | |
|---|---|---|---|---|---|
| 313 | **A** | 311 | 316 | EI**A**ETQ | 1.446 |
| 314 | **E** | 312 | 317 | IA**E**TQH | 1.136 |
| 315 | **T** | 313 | 318 | AE**T**QHG | 1.604 |
| 316 | **Q** | 314 | 319 | ET**Q**HGT | 2.291 |
| 317 | **H** | 315 | 320 | TQ**H**GTI | 0.927 |
| 318 | **G** | 316 | 321 | QH**G**TIV | 0.477 |
| 319 | **T** | 317 | 322 | HG**T**IVV | 0.204 |
| 320 | **I** | 318 | 323 | GT**I**VVR | 0.294 |
| 321 | **V** | 319 | 324 | TI**V**VRV | 0.221 |
| 322 | **V** | 320 | 325 | IV**V**RVQ | 0.265 |
| 323 | **R** | 321 | 326 | VV**R**VQY | 0.592 |
| 324 | **V** | 322 | 327 | VR**V**QYE | 1.381 |
| 325 | **Q** | 323 | 328 | RV**Q**YEG | 1.842 |
| 326 | **Y** | 324 | 329 | VQ**Y**EGD | 1.570 |
| 327 | **E** | 325 | 330 | QY**E**GDG | 2.094 |
| 328 | **G** | 326 | 331 | YE**G**DGS | 1.620 |
| 329 | **D** | 327 | 332 | EG**D**GSP | 1.599 |
| 330 | **G** | 328 | 333 | GD**G**SPC | 0.495 |
| 331 | **S** | 329 | 334 | DG**S**PCK | 1.000 |
| 332 | **P** | 330 | 335 | GS**P**CKI | 0.420 |
| 333 | **C** | 331 | 336 | SP**C**KIP | 0.656 |
| 334 | **K** | 332 | 337 | PC**K**IPF | 0.424 |
| 335 | **I** | 333 | 338 | CK**I**PFE | 0.475 |
| 336 | **P** | 334 | 339 | KI**P**FEI | 0.621 |
| 337 | **F** | 335 | 340 | IP**F**EIM | 0.307 |
| 338 | **E** | 336 | 341 | PF**E**IMD | 0.732 |
| 339 | **I** | 337 | 342 | FE**I**MDL | 0.390 |
| 340 | **M** | 338 | 343 | EI**M**DLE | 0.781 |
| 341 | **D** | 339 | 344 | IM**D**LEK | 0.901 |
| 342 | **L** | 340 | 345 | MD**L**EKR | 2.518 |
| 343 | **E** | 341 | 346 | DL**E**KRH | 3.463 |
| 344 | **K** | 342 | 347 | LE**K**RHV | 1.539 |
| 345 | **R** | 343 | 348 | EK**R**HVL | 1.539 |
| 346 | **H** | 344 | 349 | KR**H**VLG | 0.879 |
| 347 | **V** | 345 | 350 | RH**V**LGR | 0.861 |
| 348 | **L** | 346 | 351 | HV**L**GRL | 0.363 |

| | | | | | |
|---|---|---|---|---|---|
| 349 | **G** | 347 | 352 | VL**G**RLI | 0.187 |
| 350 | **R** | 348 | 353 | LG**R**LIT | 0.363 |
| 351 | **L** | 349 | 354 | GR**L**ITV | 0.327 |
| 352 | **I** | 350 | 355 | RL**I**TVN | 0.531 |
| 353 | **T** | 351 | 356 | LI**T**VNP | 0.419 |
| 354 | **V** | 352 | 357 | IT**V**NPI | 0.357 |
| 355 | **N** | 353 | 358 | TV**N**PIV | 0.377 |
| 356 | **P** | 354 | 359 | VN**P**IVT | 0.377 |
| 357 | **I** | 355 | 360 | NP**I**VTE | 0.881 |
| 358 | **V** | 356 | 361 | PI**V**TEK | 1.095 |
| 359 | **T** | 357 | 362 | IV**T**EKD | 1.183 |
| 360 | **E** | 358 | 363 | VT**E**KDS | 2.262 |
| 361 | **K** | 359 | 364 | TE**K**DSP | 4.712 |
| 362 | **D** | 360 | 365 | EK**D**SPV | 2.423 |
| 363 | **S** | 361 | 366 | KD**S**PVN | 2.250 |
| 364 | **P** | 362 | 367 | DS**P**VNI | 0.789 |
| 365 | **V** | 363 | 368 | SP**V**NIE | 0.818 |
| 366 | **N** | 364 | 369 | PV**N**IEA | 0.617 |
| 367 | **I** | 365 | 370 | VN**I**EAE | 0.691 |
| 368 | **E** | 366 | 371 | NI**E**AEP | 1.439 |
| 369 | **A** | 367 | 372 | IE**A**EPP | 1.383 |
| 370 | **E** | 368 | 373 | EA**E**PPF | 1.709 |
| 371 | **P** | 369 | 374 | AE**P**PFG | 0.976 |
| 372 | **P** | 370 | 375 | EP**P**FGD | 1.614 |
| 373 | **F** | 371 | 376 | PP**F**GDS | 1.249 |
| 374 | **G** | 372 | 377 | PF**G**DSY | 1.266 |
| 375 | **D** | 373 | 378 | FG**D**SYI | 0.574 |
| 376 | **S** | 374 | 379 | GD**S**YII | 0.464 |
| 377 | **Y** | 375 | 380 | DS**Y**III | 0.329 |
| 378 | **I** | 376 | 381 | SY**I**IIG | 0.195 |
| 379 | **I** | 377 | 382 | YI**I**IGV | 0.108 |
| 380 | **I** | 378 | 383 | II**I**GVE | 0.119 |
| 381 | **G** | 379 | 384 | II**G**VEP | 0.263 |
| 382 | **V** | 380 | 385 | IG**V**EPG | 0.372 |
| 383 | **E** | 381 | 386 | GV**E**PGQ | 0.918 |
| 384 | **P** | 382 | 387 | VE**P**GQL | 0.765 |

| | | | | | |
|---|---|---|---|---|---|
| 385 | **G** | 383 | 388 | EP**G**QLK | 2.062 |
| 386 | **Q** | 384 | 389 | PG**Q**LKL | 0.982 |
| 387 | **L** | 385 | 390 | GQ**L**KLS | 0.851 |
| 388 | **K** | 386 | 391 | QL**K**LSW | 0.904 |
| 389 | **L** | 387 | 392 | LK**L**SWF | 0.452 |
| 390 | **S** | 388 | 393 | KL**S**WFK | 1.096 |
| 391 | **W** | 389 | 394 | LS**W**FKK | 1.096 |
| 392 | **F** | 390 | 395 | SW**F**KKG | 1.315 |
| 393 | **K** | 391 | 396 | WF**K**KGS | 1.315 |
| 394 | **K** | 392 | 397 | FK**K**GSS | 1.677 |
| 395 | **G** | 393 | 398 | KK**G**SSI | 1.357 |
| 396 | **S** | 394 | 399 | KG**S**SIG | 0.672 |
| 397 | **S** | 395 | 400 | GS**S**IGQ | 0.582 |
| 398 | **I** | 396 | 401 | SS**I**GQM | 0.582 |
| 399 | **G** | 397 | 402 | SI**G**QMF | 0.376 |
| 400 | **Q** | 398 | 403 | IG**Q**MFE | 0.486 |
| 401 | **M** | 399 | 404 | GQ**M**FET | 1.000 |
| 402 | **F** | 400 | 405 | QM**F**ETT | 1.458 |
| 403 | **E** | 401 | 406 | MF**E**TTM | 0.833 |
| 404 | **T** | 402 | 407 | FE**T**TMR | 1.649 |
| 405 | **T** | 403 | 408 | ET**T**MRG | 1.885 |
| 406 | **M** | 404 | 409 | TT**M**RGA | 1.099 |
| 407 | **R** | 405 | 410 | TM**R**GAK | 1.523 |
| 408 | **G** | 406 | 411 | MR**G**AKR | 2.068 |
| 409 | **A** | 407 | 412 | RG**A**KRM | 2.068 |
| 410 | **K** | 408 | 413 | GA**K**RMA | 1.066 |
| 411 | **R** | 409 | 414 | AK**R**MAI | 0.755 |
| 412 | **M** | 410 | 415 | KR**M**AIL | 0.617 |
| 413 | **A** | 411 | 416 | RM**A**ILG | 0.305 |
| 414 | **I** | 412 | 417 | MA**I**LGD | 0.260 |
| 415 | **L** | 413 | 418 | AI**L**GDT | 0.379 |
| 416 | **G** | 414 | 419 | IL**G**DTA | 0.379 |
| 417 | **D** | 415 | 420 | LG**D**TAW | 0.569 |
| 418 | **T** | 416 | 421 | GD**T**AWD | 1.152 |
| 419 | **A** | 417 | 422 | DT**A**WDF | 1.008 |
| 420 | **W** | 418 | 423 | TA**W**DFG | 0.598 |

| | | | | | |
|---|---|---|---|---|---|
| 421 | **D** | 419 | 424 | AW**D**FGS | 0.555 |
| 422 | **F** | 420 | 425 | WD**F**GSL | 0.453 |
| 423 | **G** | 421 | 426 | DF**G**SLG | 0.426 |
| 424 | **S** | 422 | 427 | FG**S**LGG | 0.253 |
| 425 | **L** | 423 | 428 | GS**L**GGV | 0.217 |
| 426 | **G** | 424 | 429 | SL**G**GVF | 0.189 |
| 427 | **G** | 425 | 430 | LG**G**VFT | 0.204 |
| 428 | **V** | 426 | 431 | GG**V**FTS | 0.332 |
| 429 | **F** | 427 | 432 | GV**F**TSI | 0.235 |
| 430 | **T** | 428 | 433 | VF**T**SIG | 0.235 |
| 431 | **S** | 429 | 434 | FT**S**IGK | 0.633 |
| 432 | **I** | 430 | 435 | TS**I**GKA | 0.738 |
| 433 | **G** | 431 | 436 | SI**G**KAL | 0.422 |
| 434 | **K** | 432 | 437 | IG**K**ALH | 0.428 |
| 435 | **A** | 433 | 438 | GK**A**LHQ | 1.058 |
| 436 | **L** | 434 | 439 | KA**L**HQV | 0.794 |
| 437 | **H** | 435 | 440 | AL**H**QVF | 0.344 |
| 438 | **Q** | 436 | 441 | LH**Q**VFG | 0.337 |
| 439 | **V** | 437 | 442 | HQ**V**FGA | 0.412 |
| 440 | **F** | 438 | 443 | QV**F**GAI | 0.212 |
| 441 | **G** | 439 | 444 | VF**G**AIY | 0.192 |
| 442 | **A** | 440 | 445 | FG**A**IYG | 0.256 |
| 443 | **I** | 441 | 446 | GA**I**YGA | 0.299 |
| 444 | **Y** | 442 | 447 | AI**Y**GAA | 0.305 |
| 445 | **G** | 443 | 448 | IY**G**AAF | 0.262 |
| 446 | **A** | 444 | 449 | YG**A**AFS | 0.500 |
| 447 | **A** | 445 | 450 | GA**A**FSG | 0.316 |
| 448 | **F** | 446 | 451 | AA**F**SGV | 0.237 |
| 449 | **S** | 447 | 452 | AF**S**GVS | 0.314 |
| 450 | **G** | 448 | 453 | FS**G**VSW | 0.327 |
| 451 | **V** | 449 | 454 | SG**V**SWT | 0.545 |
| 452 | **S** | 450 | 455 | GV**S**WTM | 0.403 |
| 453 | **W** | 451 | 456 | VS**W**TMK | 0.814 |
| 454 | **T** | 452 | 457 | SW**T**MKI | 0.768 |
| 455 | **M** | 453 | 458 | WT**M**KIL | 0.473 |
| 456 | **K** | 454 | 459 | TM**K**ILI | 0.315 |

| | | | | | |
|---|---|---|---|---|---|
| 457 | **I** | 455 | 460 | MK**I**LIG | 0.216 |
| 458 | **L** | 456 | 461 | KI**L**IGV | 0.162 |
| 459 | **I** | 457 | 462 | IL**I**GVV | 0.060 (minimum) |
| 460 | **G** | 458 | 463 | LI**G**VVI | 0.060 (minimum) |
| 461 | **V** | 459 | 464 | IG**V**VIT | 0.105 |
| 462 | **V** | 460 | 465 | GV**V**ITW | 0.158 |
| 463 | **I** | 461 | 466 | VV**I**TWI | 0.112 |
| 464 | **T** | 462 | 467 | VI**T**WIG | 0.149 |
| 465 | **W** | 463 | 468 | IT**W**IGM | 0.199 |
| 466 | **I** | 464 | 469 | TW**I**GMN | 0.456 |
| 467 | **G** | 465 | 470 | WI**G**MNS | 0.424 |
| 468 | **M** | 466 | 471 | IG**M**NSR | 0.789 |
| 469 | **N** | 467 | 472 | GM**N**SRS | 1.509 |
| 470 | **S** | 468 | 473 | MN**S**RST | 2.201 |
| 471 | **R** | 469 | 474 | NS**R**STS | 2.980 |
| 472 | **S** | 470 | 475 | SR**S**TSL | 1.528 |
| 473 | **T** | 471 | 476 | RS**T**SLS | 1.528 |
| 474 | **S** | 472 | 477 | ST**S**LSV | 0.579 |
| 475 | **L** | 473 | 478 | TS**L**SVS | 0.579 |
| 476 | **S** | 474 | 479 | SL**S**VSL | 0.331 |
| 477 | **V** | 475 | 480 | LS**V**SLV | 0.183 |
| 478 | **S** | 476 | 481 | SV**S**LVL | 0.183 |
| 479 | **L** | 477 | 482 | VS**L**VLV | 0.102 |
| 480 | **V** | 478 | 483 | SL**V**LVG | 0.135 |
| 481 | **L** | 479 | 484 | LV**L**VGV | 0.075 |
| 482 | **V** | 480 | 485 | VL**V**GVV | 0.067 |
| 483 | **G** | 481 | 486 | LV**G**VVT | 0.131 |
| 484 | **V** | 482 | 487 | VG**V**VTL | 0.131 |
| 485 | **V** | 483 | 488 | GV**V**TLY | 0.277 |
| 486 | **T** | 484 | 489 | VV**T**LYL | 0.231 |
| 487 | **L** | 485 | 490 | VT**L**YLG | 0.308 |
| 488 | **Y** | 486 | 491 | TL**Y**LGV | 0.308 |
| 489 | **L** | 487 | 492 | LY**L**GVM | 0.211 |
| 490 | **G** | 488 | 493 | YL**G**VMV | 0.190 |
| 491 | **V** | 489 | 494 | LG**V**MVQ | 0.210 |

| | | | | | |
|---|---|---|---|---|---|
| 492 | **M** | 490 | 495 | GV**M**VQA | 0.257 |
