# Supplementary material for: Computational Prediction and Analysis of Envelop Glycoprotein Epitopes of DENV-2 and DENV-3 Pakistani Isolates: A First Step towards Dengue Vaccine Development
Source: PLoS One. 2015 Mar 16;10(3):e0119854. doi: 10.1371/journal.pone.0119854 (PMC4361635; doi:10.1371/journal.pone.0119854)
Supplement: S2 Table — (PDF) [file pone.0119854.s004.pdf]

IEDB Analysis Resource

Antibody Epitope Prediction | Example Sequences | Tutorial | External Links
Reference | Download | Contact

Emini Surface Accessibility Prediction Result Data Table

Average: 1.000 **Minimum:** 0.048 **Maximum:** 7.029 Download data to file

Position ▲ ▼	Residue	Peptide start position	Peptide end position	Peptide	Score ▲ ▼
3	**C**	1	6	MR**C**VGV	0.149
4	**V**	2	7	RC**V**GVG	0.149
5	**G**	3	8	CV**G**VGN	0.122
6	**V**	4	9	VG**V**GNR	0.446
7	**G**	5	10	GV**G**NRD	1.004
8	**N**	6	11	VG**N**RDF	0.878
9	**R**	7	12	GN**R**DFV	0.878
10	**D**	8	13	NR**D**FVE	1.537
11	**F**	9	14	RD**F**VEG	0.946
12	**V**	10	15	DF**V**EGL	0.398
13	**E**	11	16	FV**E**GLS	0.320
14	**G**	12	17	VE**G**LSG	0.365
15	**L**	13	18	EG**L**SGA	0.497
16	**S**	14	19	GL**S**GAT	0.414
17	**G**	15	20	LS**G**ATW	0.440
18	**A**	16	21	SG**A**TWV	0.396
19	**T**	17	22	GA**T**WVD	0.494
20	**W**	18	23	AT**W**VDV	0.370
21	**V**	19	24	TW**V**DVV	0.272
22	**D**	20	25	WV**D**VVL	0.155
23	**V**	21	26	VD**V**VLE	0.256
24	**V**	22	27	DV**V**LEH	0.469

25	**L**	23	28	VV**L**EHG	0.278
26	**E**	24	29	VL**E**HGG	0.371
27	**H**	25	30	LE**H**GGC	0.268
28	**G**	26	31	EH**G**GCV	0.241
29	**G**	27	32	HG**G**CVT	0.201
30	**C**	28	33	GG**C**VTT	0.213
31	**V**	29	34	GC**V**TTM	0.213
32	**T**	30	35	CV**T**TMA	0.218
33	**T**	31	36	VT**T**MAK	0.812
34	**M**	32	37	TT**M**AKN	1.758
35	**A**	33	38	TM**A**KNK	2.437
36	**K**	34	39	MA**K**NKP	2.611
37	**N**	35	40	AK**N**KPT	3.807
38	**K**	36	41	KN**K**PTL	3.108
39	**P**	37	42	NK**P**TLD	2.595
40	**T**	38	43	KP**T**LDI	1.131
41	**L**	39	44	PT**L**DIE	0.980
42	**D**	40	45	TL**D**IEL	0.523
43	**I**	41	46	LD**I**ELQ	0.627
44	**E**	42	47	DI**E**LQK	1.521
45	**L**	43	48	IE**L**QKT	1.314
46	**Q**	44	49	EL**Q**KTE	3.246
47	**K**	45	50	LQ**K**TEA	1.894
48	**T**	46	51	QK**T**EAT	3.314
49	**E**	47	52	KT**E**ATQ	3.314
50	**A**	48	53	TE**A**TQL	1.367
51	**T**	49	54	EA**T**QLA	0.957
52	**Q**	50	55	AT**Q**LAT	0.797
53	**L**	51	56	TQ**L**ATL	0.651
54	**A**	52	57	QL**A**TLR	0.883
55	**T**	53	58	LA**T**LRK	1.020
56	**L**	54	59	AT**L**RKL	1.020
57	**R**	55	60	TL**R**KLC	0.541
58	**K**	56	61	LR**K**LCI	0.263
59	**L**	57	62	RK**L**CIE	0.552
60	**C**	58	63	KL**C**IEG	0.279

61	**I**	59	64	LC**I**EGK	0.279
62	**E**	60	65	CI**E**GKI	0.237
63	**G**	61	66	IE**G**KIT	0.638
64	**K**	62	67	EG**K**ITN	1.464
65	**I**	63	68	GK**I**TNI	0.593
66	**T**	64	69	KI**T**NIT	0.864
67	**N**	65	70	IT**N**ITT	0.624
68	**I**	66	71	TN**I**TTD	1.486
69	**T**	67	72	NI**T**TDS	1.380
70	**T**	68	73	IT**T**DSR	1.680
71	**D**	69	74	TT**D**SRC	1.285
72	**S**	70	75	TD**S**RCP	1.377
73	**R**	71	76	DS**R**CPT	1.377
74	**C**	72	77	SR**C**PTQ	1.428
75	**P**	73	78	RC**P**TQG	1.054
76	**T**	74	79	CP**T**QGE	0.932
77	**Q**	75	80	PT**Q**GEA	1.757
78	**G**	76	81	TQ**G**EAV	0.843
79	**E**	77	82	QG**E**AVL	0.482
80	**A**	78	83	GE**A**VLP	0.430
81	**V**	79	84	EA**V**LPE	0.753
82	**L**	80	85	AV**L**PEE	0.753
83	**P**	81	86	VL**P**EEQ	1.291
84	**E**	82	87	LP**E**EQD	2.905
85	**E**	83	88	PE**E**QDQ	6.100
86	**Q**	84	89	EE**Q**DQN	6.344
87	**D**	85	90	EQ**D**QNY	5.739
88	**Q**	86	91	QD**Q**NYV	2.460
89	**N**	87	92	DQ**N**YVC	0.761
90	**Y**	88	93	QN**Y**VCK	0.912
91	**V**	89	94	NY**V**CKH	0.716
92	**C**	90	95	YV**C**KHT	0.643
93	**K**	91	96	VC**K**HTY	0.643
94	**H**	92	97	CK**H**TYV	0.643
95	**T**	93	98	KH**T**YVD	2.003
96	**Y**	94	99	HT**Y**VDR	1.962

97	**V**	95	100	TY**V**DRG	1.427
98	**D**	96	101	YV**D**RGW	1.039
99	**R**	97	102	VD**R**GWG	0.656
100	**G**	98	103	DR**G**WGN	1.422
101	**W**	99	104	RG**W**GNG	0.843
102	**G**	100	105	GW**G**NGC	0.231
103	**N**	101	106	WG**N**GCG	0.231
104	**G**	102	107	GN**G**CGL	0.181
105	**C**	103	108	NG**C**GLF	0.158
106	**G**	104	109	GC**G**LFG	0.097
107	**L**	105	110	CG**L**FGK	0.197
108	**F**	106	111	GL**F**GKG	0.363
109	**G**	107	112	LF**G**KGS	0.492
110	**K**	108	113	FG**K**GSL	0.492
111	**G**	109	114	GK**G**SLV	0.422
112	**S**	110	115	KG**S**LVT	0.615
113	**L**	111	116	GS**L**VTC	0.165
114	**V**	112	117	SL**V**TCA	0.168
115	**T**	113	118	LV**T**CAK	0.251
116	**C**	114	119	VT**C**AKF	0.264
117	**A**	115	120	TC**A**KFQ	0.615
118	**K**	116	121	CA**K**FQC	0.229
119	**F**	117	122	AK**F**QCL	0.352
120	**Q**	118	123	KF**Q**CLE	0.603
121	**C**	119	124	FQ**C**LEP	0.466
122	**L**	120	125	QC**L**EPI	0.377
123	**E**	121	126	CL**E**PIE	0.377
124	**P**	122	127	LE**P**IEG	0.697
125	**I**	123	128	EP**I**EGK	1.689
126	**E**	124	129	PI**E**GKV	0.724
127	**G**	125	130	IE**G**KVV	0.348
128	**K**	126	131	EG**K**VVQ	0.859
129	**V**	127	132	GK**V**VQY	0.777
130	**V**	128	133	KV**V**QYE	1.360
131	**Q**	129	134	VV**Q**YEN	1.093
132	**Y**	130	135	VQ**Y**ENL	1.215

133	**E**	131	136	QY**E**NLK	3.273
134	**N**	132	137	YE**N**LKY	2.961
135	**L**	133	138	EN**L**KYT	2.727
136	**K**	134	139	NL**K**YTV	1.169
137	**Y**	135	140	LK**Y**TVI	0.510
138	**T**	136	141	KY**T**VII	0.433
139	**V**	137	142	YT**V**IIT	0.313
140	**I**	138	143	TV**I**ITV	0.148
141	**I**	139	144	VI**I**TVH	0.140
142	**T**	140	145	II**T**VHT	0.271
143	**V**	141	146	IT**V**HTG	0.383
144	**H**	142	147	TV**H**TGD	0.913
145	**T**	143	148	VH**T**GDQ	1.095
146	**G**	144	149	HT**G**DQH	2.008
147	**D**	145	150	TG**D**QHQ	2.556
148	**Q**	146	151	GD**Q**HQV	1.315
149	**H**	147	152	DQ**H**QVG	1.315
150	**Q**	148	153	QH**Q**VGN	1.266
151	**V**	149	154	HQ**V**GNE	1.266
152	**G**	150	155	QV**G**NET	1.343
153	**N**	151	156	VG**N**ETQ	1.343
154	**E**	152	157	GN**E**TQG	1.790
155	**T**	153	158	NE**T**QGV	1.343
156	**Q**	154	159	ET**Q**GVT	1.205
157	**G**	155	160	TQ**G**VTA	0.703
158	**V**	156	161	QG**V**TAE	0.843
159	**T**	157	162	GV**T**AEI	0.341
160	**A**	158	163	VT**A**EIT	0.498
161	**E**	159	164	TA**E**ITP	1.037
162	**I**	160	165	AE**I**TPQ	1.245
163	**T**	161	166	EI**T**PQA	1.245
164	**P**	162	167	IT**P**QAS	0.963
165	**Q**	163	168	TP**Q**AST	1.983
166	**A**	164	169	PQ**A**STT	1.983
167	**S**	165	170	QA**S**TTE	2.221
168	**T**	166	171	AS**T**TEA	1.295

169	**T**	167	172	ST**T**EAI	0.899
170	**E**	168	173	TT**E**AIL	0.553
171	**A**	169	174	TE**A**ILP	0.593
172	**I**	170	175	EA**I**LPE	0.711
173	**L**	171	176	AI**L**PEY	0.643
174	**P**	172	177	IL**P**EYG	0.630
175	**E**	173	178	LP**E**YGT	1.298
176	**Y**	174	179	PE**Y**GTL	1.298
177	**G**	175	180	EY**G**TLG	0.831
178	**T**	176	181	YG**T**LGL	0.396
179	**L**	177	182	GT**L**GLE	0.437
180	**G**	178	183	TL**G**LEC	0.237
181	**L**	179	184	LG**L**ECS	0.220
182	**E**	180	185	GL**E**CSP	0.412
183	**C**	181	186	LE**C**SPR	0.816
184	**S**	182	187	EC**S**PRT	1.428
185	**P**	183	188	CS**P**RTG	0.816
186	**R**	184	189	SP**R**TGL	1.255
187	**T**	185	190	PR**T**GLD	1.564
188	**G**	186	191	RT**G**LDF	0.876
189	**L**	187	192	TG**L**DFN	0.719
190	**D**	188	193	GL**D**FNE	0.863
191	**F**	189	194	LD**F**NEM	0.863
192	**N**	190	195	DF**N**EMI	0.734
193	**E**	191	196	FN**E**MIL	0.362
194	**M**	192	197	NE**M**ILL	0.345
195	**I**	193	198	EM**I**LLT	0.310
196	**L**	194	199	MI**L**LTM	0.177
197	**L**	195	200	IL**L**TMK	0.358
198	**T**	196	201	LL**T**MKN	0.820
199	**M**	197	202	LT**M**KNK	1.989
200	**K**	198	203	TM**K**NKA	2.437
201	**N**	199	204	MK**N**KAW	1.775
202	**K**	200	205	KN**K**AWM	1.775
203	**A**	201	206	NK**A**WMV	0.659
204	**W**	202	207	KA**W**MVH	0.558

205	**M**	203	208	AW**M**VHR	0.546
206	**V**	204	209	WM**V**HRQ	0.936
207	**H**	205	210	MV**H**RQW	0.936
208	**R**	206	211	VH**R**QWF	0.819
209	**Q**	207	212	HR**Q**WFF	0.956
210	**W**	208	213	RQ**W**FFD	1.173
211	**F**	209	214	QW**F**FDL	0.494
212	**F**	210	215	WF**F**DLP	0.441
213	**D**	211	216	FF**D**LPL	0.346
214	**L**	212	217	FD**L**PLP	0.617
215	**P**	213	218	DL**P**LPW	0.750
216	**L**	214	219	LP**L**PWT	0.648
217	**P**	215	220	PL**P**WTS	1.053
218	**W**	216	221	LP**W**TSG	0.674
219	**T**	217	222	PW**T**SGA	0.826
220	**S**	218	223	WT**S**GAT	0.770
221	**G**	219	224	TS**G**ATT	1.058
222	**A**	220	225	SG**A**TTE	1.269
223	**T**	221	226	GA**T**TET	1.367
224	**T**	222	227	AT**T**ETP	2.135
225	**E**	223	228	TT**E**TPT	3.051
226	**T**	224	229	TE**T**PTW	2.223
227	**P**	225	230	ET**P**TWN	2.477
228	**T**	226	231	TP**T**WNR	2.801
229	**W**	227	232	PT**W**NRK	3.881
230	**N**	228	233	TW**N**RKE	4.347
231	**R**	229	234	WN**R**KEL	2.484
232	**K**	230	235	NR**K**ELL	1.948
233	**E**	231	236	RK**E**LLV	0.899
234	**L**	232	237	KE**L**LVT	0.663
235	**L**	233	238	EL**L**VTF	0.287
236	**V**	234	239	LL**V**TFK	0.331
237	**T**	235	240	LV**T**FKN	0.646
238	**F**	236	241	VT**F**KNA	0.791
239	**K**	237	242	TF**K**NAH	1.451
240	**N**	238	243	FK**N**AHA	1.016

241	**A**	239	244	KN**A**HAK	2.345
242	**H**	240	245	NA**H**AKK	2.345
243	**A**	241	246	AH**A**KKQ	2.526
244	**K**	242	247	HA**K**KQE	4.330
245	**K**	243	248	AK**K**QEV	2.362
246	**Q**	244	249	KK**Q**EVV	1.735
247	**E**	245	250	KQ**E**VVV	0.644
248	**V**	246	251	QE**V**VVL	0.266
249	**V**	247	252	EV**V**VLG	0.152
250	**V**	248	253	VV**V**LGS	0.117
251	**L**	249	254	VV**L**GSQ	0.274
252	**G**	250	255	VL**G**SQE	0.639
253	**S**	251	256	LG**S**QEG	0.852
254	**Q**	252	257	GS**Q**EGA	1.044
255	**E**	253	258	SQ**E**GAM	1.044
256	**G**	254	259	QE**G**AMH	1.060
257	**A**	255	260	EG**A**MHT	0.884
258	**M**	256	261	GA**M**HTA	0.515
259	**H**	257	262	AM**H**TAL	0.430
260	**T**	258	263	MH**T**ALT	0.614
261	**A**	259	264	HT**A**LTG	0.614
262	**L**	260	265	TA**L**TGA	0.456
263	**T**	261	266	AL**T**GAT	0.456
264	**G**	262	267	LT**G**ATE	0.781
265	**A**	263	268	TG**A**TEI	0.664
266	**T**	264	269	GA**T**EIQ	0.797
267	**E**	265	270	AT**E**IQN	1.294
268	**I**	266	271	TE**I**QNS	1.717
269	**Q**	267	272	EI**Q**NSG	1.177
270	**N**	268	273	IQ**N**SGG	0.673
271	**S**	269	274	QN**S**GGT	1.385
272	**G**	270	275	NS**G**GTS	1.072
273	**G**	271	276	SG**G**TSI	0.467
274	**T**	272	277	GG**T**SIF	0.302
275	**S**	273	278	GT**S**IFA	0.308
276	**I**	274	279	TS**I**FAG	0.308

277	**F**	275	280	SI**F**AGH	0.291
278	**A**	276	281	IF**A**GHL	0.179
279	**G**	277	282	FA**G**HLK	0.510
280	**H**	278	283	AG**H**LKC	0.316
281	**L**	279	284	GH**L**KCR	0.612
282	**K**	280	285	HL**K**CRL	0.510
283	**C**	281	286	LK**C**RLK	0.750
284	**R**	282	287	KC**R**LKM	0.900
285	**L**	283	288	CR**L**KMD	0.751
286	**K**	284	289	RL**K**MDK	2.803
287	**M**	285	290	LK**M**DKL	1.180
288	**D**	286	291	KM**D**KLE	2.479
289	**K**	287	292	MD**K**LEL	1.022
290	**L**	288	293	DK**L**ELK	2.066
291	**E**	289	294	KL**E**LKG	1.224
292	**L**	290	295	LE**L**KGM	0.606
293	**K**	291	296	EL**K**GMS	0.984
294	**G**	292	297	LK**G**MSY	0.891
295	**M**	293	298	KG**M**SYA	1.091
296	**S**	294	299	GM**S**YAM	0.540
297	**Y**	295	300	MS**Y**AMC	0.292
298	**A**	296	301	SY**A**MCT	0.426
299	**M**	297	302	YA**M**CTN	0.512
300	**C**	298	303	AM**C**TNT	0.471
301	**T**	299	304	MC**T**NTF	0.404
302	**N**	300	305	CT**N**TFV	0.303
303	**T**	301	306	TN**T**FVL	0.466
304	**F**	302	307	NT**F**VLK	0.646
305	**V**	303	308	TF**V**LKK	0.803
306	**L**	304	309	FV**L**KKE	0.964
307	**K**	305	310	VL**K**KEV	0.826
308	**K**	306	311	LK**K**EVS	1.492
309	**E**	307	312	KK**E**VSE	3.133
310	**V**	308	313	KE**V**SET	2.261
311	**S**	309	314	EV**S**ETQ	1.958
312	**E**	310	315	VS**E**TQH	1.538

313	**T**	311	316	SE**T**QHG	2.051
314	**Q**	312	317	ET**Q**HGT	2.209
315	**H**	313	318	TQ**H**GTI	0.894
316	**G**	314	319	QH**G**TIL	0.511
317	**T**	315	320	HG**T**ILI	0.207
318	**I**	316	321	GT**I**LIK	0.304
319	**L**	317	322	TI**L**IKV	0.228
320	**I**	318	323	IL**I**KVE	0.274
321	**K**	319	324	LI**K**VEY	0.611
322	**V**	320	325	IK**V**EYK	1.483
323	**E**	321	326	KV**E**YKG	2.093
324	**Y**	322	327	VE**Y**KGE	1.813
325	**K**	323	328	EY**K**GED	4.079
326	**G**	324	329	YK**G**EDA	2.379
327	**E**	325	330	KG**E**DAP	2.348
328	**D**	326	331	GE**D**APC	0.629
329	**A**	327	332	ED**A**PCK	1.272
330	**P**	328	333	DA**P**CKI	0.515
331	**C**	329	334	AP**C**KIP	0.477
332	**K**	330	335	PC**K**IPF	0.409
333	**I**	331	336	CK**I**PFS	0.354
334	**P**	332	337	KI**P**FST	0.953
335	**F**	333	338	IP**F**STE	0.826
336	**S**	334	339	PF**S**TED	1.967
337	**T**	335	340	FS**T**EDG	1.259
338	**E**	336	341	ST**E**DGQ	2.517
339	**D**	337	342	TE**D**GQG	1.859
340	**G**	338	343	ED**G**QGK	2.576
341	**Q**	339	344	DG**Q**GKA	1.503
342	**G**	340	345	GQ**G**KAH	1.224
343	**K**	341	346	QG**K**AHN	1.990
344	**A**	342	347	GK**A**HNG	1.137
345	**H**	343	348	KA**H**NGR	2.250
346	**N**	344	349	AH**N**GRL	0.928
347	**G**	345	350	HN**G**RLI	0.644
348	**R**	346	351	NG**R**LIT	0.683

349	**L**	347	352	GR**L**ITA	0.429
350	**I**	348	353	RL**I**TAN	0.697
351	**T**	349	354	LI**T**ANP	0.550
352	**A**	350	355	IT**A**NPV	0.495
353	**N**	351	356	TA**N**PVV	0.524
354	**P**	352	357	AN**P**VVT	0.524
355	**V**	353	358	NP**V**VTK	1.038
356	**V**	354	359	PV**V**TKK	1.291
357	**T**	355	360	VV**T**KKE	1.446
358	**K**	356	361	VT**K**KEE	3.374
359	**K**	357	362	TK**K**EEP	7.029 (maximum)
360	**E**	358	363	KK**E**EPV	3.615
361	**E**	359	364	KE**E**PVN	2.907
362	**P**	360	365	EE**P**VNI	1.019
363	**V**	361	366	EP**V**NIE	1.019
364	**N**	362	367	PV**N**IEA	0.594
365	**I**	363	368	VN**I**EAE	0.666
366	**E**	364	369	NI**E**AEP	1.387
367	**A**	365	370	IE**A**EPP	1.334
368	**E**	366	371	EA**E**PPF	1.647
369	**P**	367	372	AE**P**PFG	0.941
370	**P**	368	373	EP**P**FGE	1.614
371	**F**	369	374	PP**F**GES	1.249
372	**G**	370	375	PF**G**ESN	1.299
373	**E**	371	376	FG**E**SNI	0.589
374	**S**	372	377	GE**S**NIV	0.505
375	**N**	373	378	ES**N**IVI	0.357
376	**I**	374	379	SN**I**VIG	0.204
377	**V**	375	380	NI**V**IGI	0.107
378	**I**	376	381	IV**I**GIG	0.066
379	**G**	377	382	VI**G**IGD	0.157
380	**I**	378	383	IG**I**GDN	0.339
381	**G**	379	384	GI**G**DNA	0.489
382	**D**	380	385	IG**D**NAL	0.408
383	**N**	381	386	GD**N**ALK	1.163

384	**A**	382	387	DN**A**LKI	0.824
385	**L**	383	388	NA**L**KIN	0.793
386	**K**	384	389	AL**K**INW	0.519
387	**I**	385	390	LK**I**NWY	0.804
388	**N**	386	391	KI**N**WYK	1.950
389	**W**	387	392	IN**W**YKK	1.950
390	**Y**	388	393	NW**Y**KKG	2.754
391	**K**	389	394	WY**K**KGS	2.295
392	**K**	390	395	YK**K**GSS	2.925
393	**G**	391	396	KK**G**SSI	1.308
394	**S**	392	397	KG**S**SIG	0.647
395	**S**	393	398	GS**S**IGK	0.647
396	**I**	394	399	SS**I**GKM	0.647
397	**G**	395	400	SI**G**KMF	0.418
398	**K**	396	401	IG**K**MFE	0.541
399	**M**	397	402	GK**M**FEA	0.779
400	**F**	398	403	KM**F**EAT	1.136
401	**E**	399	404	MF**E**ATA	0.574
402	**A**	400	405	FE**A**TAR	1.136
403	**T**	401	406	EA**T**ARG	1.298
404	**A**	402	407	AT**A**RGA	0.757
405	**R**	403	408	TA**R**GAR	1.468
406	**G**	404	409	AR**G**ARR	1.993
407	**A**	405	410	RG**A**RRM	1.952
408	**R**	406	411	GA**R**RMA	1.007
409	**R**	407	412	AR**R**MAI	0.713
410	**M**	408	413	RR**M**AIL	0.582
411	**A**	409	414	RM**A**ILG	0.294
412	**I**	410	415	MA**I**LGD	0.251
413	**L**	411	416	AI**L**GDT	0.366
414	**G**	412	417	IL**G**DTA	0.366
415	**D**	413	418	LG**D**TAW	0.549
416	**T**	414	419	GD**T**AWD	1.111
417	**A**	415	420	DT**A**WDF	0.972
418	**W**	416	421	TA**W**DFG	0.576
419	**D**	417	422	AW**D**FGS	0.535

420	**F**	418	423	WD**F**GSV	0.393
421	**G**	419	424	DF**G**SVG	0.370
422	**S**	420	425	FG**S**VGG	0.219
423	**V**	421	426	GS**V**GGV	0.188
424	**G**	422	427	SV**G**GVL	0.157
425	**G**	423	428	VG**G**VLN	0.188
426	**V**	424	429	GG**V**LNS	0.339
427	**L**	425	430	GV**L**NSL	0.283
428	**N**	426	431	VL**N**SLG	0.283
429	**S**	427	432	LN**S**LGK	0.762
430	**L**	428	433	NS**L**GKM	0.914
431	**G**	429	434	SL**G**KMV	0.422
432	**K**	430	435	LG**K**MVH	0.428
433	**M**	431	436	GK**M**VHQ	0.900
434	**V**	432	437	KM**V**HQI	0.637
435	**H**	433	438	MV**H**QIF	0.276
436	**Q**	434	439	VH**Q**IFG	0.276
437	**I**	435	440	HQ**I**FGS	0.498
438	**F**	436	441	QI**F**GSA	0.370
439	**G**	437	442	IF**G**SAY	0.335
440	**S**	438	443	FG**S**AYT	0.689
441	**A**	439	444	GS**A**YTA	0.804
442	**Y**	440	445	SA**Y**TAL	0.670
443	**T**	441	446	AY**T**ALF	0.433
444	**A**	442	447	YT**A**LFS	0.574
445	**L**	443	448	TA**L**FSG	0.363
446	**F**	444	449	AL**F**SGV	0.186
447	**S**	445	450	LF**S**GVS	0.247
448	**G**	446	451	FS**G**VSW	0.315
449	**V**	447	452	SG**V**SWV	0.270
450	**S**	448	453	GV**S**WVM	0.200
451	**W**	449	454	VS**W**VMK	0.403
452	**V**	450	455	SW**V**MKI	0.381
453	**M**	451	456	WV**M**KIG	0.281
454	**K**	452	457	VM**K**IGI	0.188
455	**I**	453	458	MK**I**GIG	0.250

456	**G**	454	459	KI**G**IGV	0.188
457	**I**	455	460	IG**I**GVL	0.077
458	**G**	456	461	GI**G**VLL	0.091
459	**V**	457	462	IG**V**LLT	0.133
460	**L**	458	463	GV**L**LTW	0.199
461	**L**	459	464	VL**L**TWI	0.141
462	**T**	460	465	LL**T**WIG	0.188
463	**W**	461	466	LT**W**IGL	0.188
464	**I**	462	467	TW**I**GLN	0.367
465	**G**	463	468	WI**G**LNS	0.340
466	**L**	464	469	IG**L**NSK	0.647
467	**N**	465	470	GL**N**SKN	1.485
468	**S**	466	471	LN**S**KNT	2.166
469	**K**	467	472	NS**K**NTS	3.520
470	**N**	468	473	SK**N**TSM	2.166
471	**T**	469	474	KN**T**SMS	2.166
472	**S**	470	475	NT**S**MSF	0.938
473	**M**	471	476	TS**M**SFS	0.782
474	**S**	472	477	SM**S**FSC	0.290
475	**F**	473	478	MS**F**SCI	0.152
476	**S**	474	479	SF**S**CIA	0.155
477	**C**	475	480	FS**C**IAI	0.081
478	**I**	476	481	SC**I**AIG	0.093
479	**A**	477	482	CI**A**IGI	0.048 (minimum)
480	**I**	478	483	IA**I**GII	0.063
481	**G**	479	484	AI**G**IIT	0.131
482	**I**	480	485	IG**I**ITL	0.107
483	**I**	481	486	GI**I**TLY	0.238
484	**T**	482	487	II**T**LYL	0.198
485	**L**	483	488	IT**L**YLG	0.280
486	**Y**	484	489	TL**Y**LGA	0.404
487	**L**	485	490	LY**L**GAV	0.208
488	**G**	486	491	YL**G**AVV	0.187
489	**A**	487	492	LG**A**VVQ	0.207
490	**V**	488	493	GA**V**VQA	0.253
