# Supplementary material for: Computational Prediction and Analysis of Envelop Glycoprotein Epitopes of DENV-2 and DENV-3 Pakistani Isolates: A First Step towards Dengue Vaccine Development
Source: PLoS One. 2015 Mar 16;10(3):e0119854. doi: 10.1371/journal.pone.0119854 (PMC4361635; doi:10.1371/journal.pone.0119854)
Supplement: S3 Table — (PDF) [file pone.0119854.s005.pdf]

IEDB Analysis Resource

Antibody Epitope Prediction | Example Sequences | Tutorial | External Links
Reference | Download | Contact

Karplus & Schulz Flexibility Prediction Result Data Table

Average: 0.996 **Minimum:** 0.884 **Maximum:** 1.108 Download data to file

Position ▲ ▼	Residue	Peptide start position	Peptide end position	Peptide	Score ▲ ▼
4	**I**	1	7	MRC**I**GIS	0.923
5	**G**	2	8	RCI**G**ISN	0.953
6	**I**	3	9	CIG**I**SNR	0.991
7	**S**	4	10	IGI**S**NRD	1.028
8	**N**	5	11	GIS**N**RDF	1.046
9	**R**	6	12	ISN**R**DFV	1.039
10	**D**	7	13	SNR**D**FVE	1.024
11	**F**	8	14	NRD**F**VEG	1.000
12	**V**	9	15	RDF**V**EGV	0.990
13	**E**	10	16	DFV**E**GVS	0.999
14	**G**	11	17	FVE**G**VSG	1.014
15	**V**	12	18	VEG**V**SGG	1.039
16	**S**	13	19	EGV**S**GGS	1.064
17	**G**	14	20	GVS**G**GSW	1.072
18	**G**	15	21	VSG**G**SWV	1.061
19	**S**	16	22	SGG**S**WVD	1.030
20	**W**	17	23	GGS**W**VDI	0.986
21	**V**	18	24	GSW**V**DIV	0.949
22	**D**	19	25	SWV**D**IVL	0.928
23	**I**	20	26	WVD**I**VLE	0.921
24	**V**	21	27	VDI**V**LEH	0.928
25	**L**	22	28	DIV**L**EHG	0.944

26	**E**	23	29	IVL**E**HGS	0.963
27	**H**	24	30	VLE**H**GSC	0.979
28	**G**	25	31	LEH**G**SCV	0.985
29	**S**	26	32	EHG**S**CVT	0.985
30	**C**	27	33	HGS**C**VTT	0.978
31	**V**	28	34	GSC**V**TTM	0.975
32	**T**	29	35	SCV**T**TMA	0.976
33	**T**	30	36	CVT**T**MAK	0.978
34	**M**	31	37	VTT**M**AKN	0.984
35	**A**	32	38	TTM**A**KNK	1.006
36	**K**	33	39	TMA**K**NKP	1.033
37	**N**	34	40	MAK**N**KPT	1.057
38	**K**	35	41	AKN**K**PTL	1.067
39	**P**	36	42	KNK**P**TLD	1.052
40	**T**	37	43	NKP**T**LDF	1.023
41	**L**	38	44	KPT**L**DFE	0.988
42	**D**	39	45	PTL**D**FEL	0.963
43	**F**	40	46	TLD**F**ELI	0.945
44	**E**	41	47	LDF**E**LIK	0.944
45	**L**	42	48	DFE**L**IKT	0.959
46	**I**	43	49	FEL**I**KTE	0.980
47	**K**	44	50	ELI**K**TEA	1.012
48	**T**	45	51	LIK**T**EAK	1.035
49	**E**	46	52	IKT**E**AKQ	1.054
50	**A**	47	53	KTE**A**KQP	1.073
51	**K**	48	54	TEA**K**QPA	1.081
52	**Q**	49	55	EAK**Q**PAT	1.081
53	**P**	50	56	AKQ**P**ATL	1.062
54	**A**	51	57	KQP**A**TLR	1.037
55	**T**	52	58	QPA**T**LRK	1.015
56	**L**	53	59	PAT**L**RKY	1.001
57	**R**	54	60	ATL**R**KYC	0.997
58	**K**	55	61	TLR**K**YCI	0.988
59	**Y**	56	62	LRK**Y**CIE	0.968
60	**C**	57	63	RKY**C**IEA	0.954
61	**I**	58	64	KYC**I**EAK	0.951

62	**E**	59	65	YCI**E**AKL	0.962
63	**A**	60	66	CIE**A**KLT	0.989
64	**K**	61	67	IEA**K**LTN	1.014
65	**L**	62	68	EAK**L**TNT	1.032
66	**T**	63	69	AKL**T**NTT	1.052
67	**N**	64	70	KLT**N**TTT	1.064
68	**T**	65	71	LTN**T**TTA	1.067
69	**T**	66	72	TNT**T**TAS	1.067
70	**T**	67	73	NTT**T**ASR	1.058
71	**A**	68	74	TTT**A**SRC	1.044
72	**S**	69	75	TTA**S**RCP	1.036
73	**R**	70	76	TAS**R**CPT	1.032
74	**C**	71	77	ASR**C**PTQ	1.039
75	**P**	72	78	SRC**P**TQG	1.063
76	**T**	73	79	RCP**T**QGE	1.086
77	**Q**	74	80	CPT**Q**GEP	1.105
78	**G**	75	81	PTQ**G**EPS	1.108 (maximum)
79	**E**	76	82	TQG**E**PSL	1.090
80	**P**	77	83	QGE**P**SLN	1.065
81	**S**	78	84	GEP**S**LNE	1.043
82	**L**	79	85	EPS**L**NEE	1.032
83	**N**	80	86	PSL**N**EEQ	1.044
84	**E**	81	87	SLN**E**EQD	1.064
85	**E**	82	88	LNE**E**QDK	1.081
86	**Q**	83	89	NEE**Q**DKR	1.091
87	**D**	84	90	EEQ**D**KRF	1.072
88	**K**	85	91	EQD**K**RFV	1.045
89	**R**	86	92	QDK**R**FVC	1.004
90	**F**	87	93	DKR**F**VCK	0.969
91	**V**	88	94	KRF**V**CKH	0.954
92	**C**	89	95	RFV**C**KHS	0.948
93	**K**	90	96	FVC**K**HSM	0.953
94	**H**	91	97	VCK**H**SMV	0.949
95	**S**	92	98	CKH**S**MVD	0.947
96	**M**	93	99	KHS**M**VDR	0.952
97	**V**	94	100	HSM**V**DRG	0.969

98	**D**	95	101	SMV**D**RGW	0.994
99	**R**	96	102	MVD**R**GWG	1.010
100	**G**	97	103	VDR**G**WGN	1.023
101	**W**	98	104	DRG**W**GNG	1.025
102	**G**	99	105	RGW**G**NGC	1.029
103	**N**	100	106	GWG**N**GCG	1.028
104	**G**	101	107	WGN**G**CGL	1.013
105	**C**	102	108	GNG**C**GLF	0.990
106	**G**	103	109	NGC**G**LFG	0.970
107	**L**	104	110	GCG**L**FGK	0.968
108	**F**	105	111	CGL**F**GKG	0.987
109	**G**	106	112	GLF**G**KGG	1.022
110	**K**	107	113	LFG**K**GGI	1.043
111	**G**	108	114	FGK**G**GIV	1.043
112	**G**	109	115	GKG**G**IVT	1.018
113	**I**	110	116	KGG**I**VTC	0.975
114	**V**	111	117	GGI**V**TCA	0.941
115	**T**	112	118	GIV**T**CAM	0.910
116	**C**	113	119	IVT**C**AMF	0.891
117	**A**	114	120	VTC**A**MFT	0.885
118	**M**	115	121	TCA**M**FTC	0.884 (minimum)
119	**F**	116	122	CAM**F**TCK	0.908
120	**T**	117	123	AMF**T**CKK	0.944
121	**C**	118	124	MFT**C**KKN	0.982
122	**K**	119	125	FTC**K**KNM	1.014
123	**K**	120	126	TCK**K**NME	1.026
124	**N**	121	127	CKK**N**MEG	1.030
125	**M**	122	128	KKN**M**EGK	1.034
126	**E**	123	129	KNM**E**GKI	1.036
127	**G**	124	130	NME**G**KIV	1.033
128	**K**	125	131	MEG**K**IVQ	1.021
129	**I**	126	132	EGK**I**VQP	1.002
130	**V**	127	133	GKI**V**QPE	0.999
131	**Q**	128	134	KIV**Q**PEN	1.007
132	**P**	129	135	IVQ**P**ENL	1.019

133	**E**	130	136	VQP**E**NLE	1.026
134	**N**	131	137	QPE**N**LEY	1.011
135	**L**	132	138	PEN**L**EYT	0.986
136	**E**	133	139	ENL**E**YTI	0.961
137	**Y**	134	140	NLE**Y**TIV	0.943
138	**T**	135	141	LEY**T**IVV	0.930
139	**I**	136	142	EYT**I**VVT	0.929
140	**V**	137	143	YTI**V**VTP	0.945
141	**V**	138	144	TIV**V**TPH	0.967
142	**T**	139	145	IVV**T**PHS	0.998
143	**P**	140	146	VVT**P**HSG	1.028
144	**H**	141	147	VTP**H**SGE	1.046
145	**S**	142	148	TPH**S**GEE	1.067
146	**G**	143	149	PHS**G**EEN	1.077
147	**E**	144	150	HSG**E**ENA	1.070
148	**E**	145	151	SGE**E**NAV	1.052
149	**N**	146	152	GEE**N**AVG	1.023
150	**A**	147	153	EEN**A**VGN	1.004
151	**V**	148	154	ENA**V**GND	1.003
152	**G**	149	155	NAV**G**NDT	1.021
153	**N**	150	156	AVG**N**DTG	1.049
154	**D**	151	157	VGN**D**TGK	1.067
155	**T**	152	158	GND**T**GKH	1.077
156	**G**	153	159	NDT**G**KHG	1.076
157	**K**	154	160	DTG**K**HGK	1.066
158	**H**	155	161	TGK**H**GKE	1.055
159	**G**	156	162	GKH**G**KEI	1.048
160	**K**	157	163	KHG**K**EIK	1.045
161	**E**	158	164	HGK**E**IKV	1.037
162	**I**	159	165	GKE**I**KVT	1.033
163	**K**	160	166	KEI**K**VTP	1.031
164	**V**	161	167	EIK**V**TPQ	1.035
165	**T**	162	168	IKV**T**PQS	1.058
166	**P**	163	169	KVT**P**QSS	1.080
167	**Q**	164	170	VTP**Q**SSI	1.095
168	**S**	165	171	TPQ**S**SIT	1.096

169	**S**	166	172	PQS**S**ITE	1.077
170	**I**	167	173	QSS**I**TEA	1.058
171	**T**	168	174	SSI**T**EAE	1.037
172	**E**	169	175	SIT**E**AEL	1.019
173	**A**	170	176	ITE**A**ELT	1.010
174	**E**	171	177	TEA**E**LTG	1.001
175	**L**	172	178	EAE**L**TGY	0.999
176	**T**	173	179	AEL**T**GYG	1.006
177	**G**	174	180	ELT**G**YGT	1.013
178	**Y**	175	181	LTG**Y**GTV	1.016
179	**G**	176	182	TGY**G**TVT	1.015
180	**T**	177	183	GYG**T**VTM	1.003
181	**V**	178	184	YGT**V**TME	0.985
182	**T**	179	185	GTV**T**MEC	0.968
183	**M**	180	186	TVT**M**ECS	0.961
184	**E**	181	187	VTM**E**CSP	0.966
185	**C**	182	188	TME**C**SPR	0.985
186	**S**	183	189	MEC**S**PRT	1.012
187	**P**	184	190	ECS**P**RTG	1.032
188	**R**	185	191	CSP**R**TGL	1.040
189	**T**	186	192	SPR**T**GLD	1.035
190	**G**	187	193	PRT**G**LDF	1.013
191	**L**	188	194	RTG**L**DFN	0.990
192	**D**	189	195	TGL**D**FNE	0.976
193	**F**	190	196	GLD**F**NEM	0.965
194	**N**	191	197	LDF**N**EMV	0.962
195	**E**	192	198	DFN**E**MVL	0.955
196	**M**	193	199	FNE**M**VLL	0.940
197	**V**	194	200	NEM**V**LLQ	0.930
198	**L**	195	201	EMV**L**LQM	0.924
199	**L**	196	202	MVL**L**QME	0.930
200	**Q**	197	203	VLL**Q**MEN	0.951
201	**M**	198	204	LLQ**M**ENK	0.982
202	**E**	199	205	LQM**E**NKA	1.011
203	**N**	200	206	QME**N**KAW	1.018
204	**K**	201	207	MEN**K**AWL	1.004

205	**A**	202	208	ENK**A**WLV	0.967
206	**W**	203	209	NKA**W**LVH	0.930
207	**L**	204	210	KAW**L**VHR	0.921
208	**V**	205	211	AWL**V**HRQ	0.932
209	**H**	206	212	WLV**H**RQW	0.953
210	**R**	207	213	LVH**R**QWF	0.972
211	**Q**	208	214	VHR**Q**WFL	0.972
212	**W**	209	215	HRQ**W**FLD	0.960
213	**F**	210	216	RQW**F**LDL	0.950
214	**L**	211	217	QWF**L**DLP	0.944
215	**D**	212	218	WFL**D**LPL	0.943
216	**L**	213	219	FLD**L**PLP	0.948
217	**P**	214	220	LDL**P**LPW	0.946
218	**L**	215	221	DLP**L**PWL	0.945
219	**P**	216	222	LPL**P**WLP	0.951
220	**W**	217	223	PLP**W**LPG	0.964
221	**L**	218	224	LPW**L**PGA	0.988
222	**P**	219	225	PWL**P**GAD	1.008
223	**G**	220	226	WLP**G**ADI	1.016
224	**A**	221	227	LPG**A**DIQ	1.016
225	**D**	222	228	PGA**D**IQG	1.014
226	**I**	223	229	GAD**I**QGS	1.029
227	**Q**	224	230	ADI**Q**GSN	1.050
228	**G**	225	231	DIQ**G**SNW	1.064
229	**S**	226	232	IQG**S**NWI	1.056
230	**N**	227	233	QGS**N**WIQ	1.025
231	**W**	228	234	GSN**W**IQK	0.999
232	**I**	229	235	SNW**I**QKE	0.991
233	**Q**	230	236	NWI**Q**KET	1.009
234	**K**	231	237	WIQ**K**ETL	1.031
235	**E**	232	238	IQK**E**TLV	1.038
236	**T**	233	239	QKE**T**LVT	1.023
237	**L**	234	240	KET**L**VTF	0.991
238	**V**	235	241	ETL**V**TFK	0.971
239	**T**	236	242	TLV**T**FKN	0.973
240	**F**	237	243	LVT**F**KNP	0.994

241	**K**	238	244	VTF**K**NPH	1.024
242	**N**	239	245	TFK**N**PHA	1.038
243	**P**	240	246	FKN**P**HAK	1.038
244	**H**	241	247	KNP**H**AKK	1.030
245	**A**	242	248	NPH**A**KKQ	1.033
246	**K**	243	249	PHA**K**KQD	1.053
247	**K**	244	250	HAK**K**QDV	1.066
248	**Q**	245	251	AKK**Q**DVV	1.068
249	**D**	246	252	KKQ**D**VVV	1.040
250	**V**	247	253	KQD**V**VVL	0.995
251	**V**	248	254	QDV**V**VLG	0.967
252	**V**	249	255	DVV**V**LGS	0.967
253	**L**	250	256	VVV**L**GSQ	0.998
254	**G**	251	257	VVL**G**SQE	1.045
255	**S**	252	258	VLG**S**QEG	1.085
256	**Q**	253	259	LGS**Q**EGA	1.095
257	**E**	254	260	GSQ**E**GAM	1.067
258	**G**	255	261	SQE**G**AMH	1.022
259	**A**	256	262	QEG**A**MHT	0.970
260	**M**	257	263	EGA**M**HTA	0.931
261	**H**	258	264	GAM**H**TAL	0.926
262	**T**	259	265	AMH**T**ALT	0.935
263	**A**	260	266	MHT**A**LTG	0.958
264	**L**	261	267	HTA**L**TGA	0.986
265	**T**	262	268	TAL**T**GAT	1.011
266	**G**	263	269	ALT**G**ATE	1.028
267	**A**	264	270	LTG**A**TEI	1.036
268	**T**	265	271	TGA**T**EIQ	1.029
269	**E**	266	272	GAT**E**IQM	1.009
270	**I**	267	273	ATE**I**QMS	0.991
271	**Q**	268	274	TEI**Q**MSS	0.986
272	**M**	269	275	EIQ**M**SSG	1.007
273	**S**	270	276	IQM**S**SGN	1.038
274	**S**	271	277	QMS**S**GNL	1.062
275	**G**	272	278	MSS**G**NLL	1.061
276	**N**	273	279	SSG**N**LLF	1.030

277	**L**	274	280	SGN**L**LFT	0.997
278	**L**	275	281	GNL**L**FTG	0.975
279	**F**	276	282	NLL**F**TGH	0.970
280	**T**	277	283	LLF**T**GHL	0.983
281	**G**	278	284	LFT**G**HLK	0.994
282	**H**	279	285	FTG**H**LKC	0.994
283	**L**	280	286	TGH**L**KCR	0.989
284	**K**	281	287	GHL**K**CRL	0.982
285	**C**	282	288	HLK**C**RLR	0.964
286	**R**	283	289	LKC**R**LRM	0.951
287	**L**	284	290	KCR**L**RMD	0.952
288	**R**	285	291	CRL**R**MDK	0.960
289	**M**	286	292	RLR**M**DKL	0.983
290	**D**	287	293	LRM**D**KLQ	1.002
291	**K**	288	294	RMD**K**LQL	1.002
292	**L**	289	295	MDK**L**QLK	0.994
293	**Q**	290	296	DKL**Q**LKG	0.988
294	**L**	291	297	KLQ**L**KGM	0.988
295	**K**	292	298	LQL**K**GMS	0.994
296	**G**	293	299	QLK**G**MSY	0.993
297	**M**	294	300	LKG**M**SYS	0.979
298	**S**	295	301	KGM**S**YSM	0.956
299	**Y**	296	302	GMS**Y**SMC	0.932
300	**S**	297	303	MSY**S**MCT	0.923
301	**M**	298	304	SYS**M**CTG	0.934
302	**C**	299	305	YSM**C**TGK	0.964
303	**T**	300	306	SMC**T**GKF	1.000
304	**G**	301	307	MCT**G**KFK	1.031
305	**K**	302	308	CTG**K**FKV	1.035
306	**F**	303	309	TGK**F**KVV	1.016
307	**K**	304	310	GKF**K**VVK	1.003
308	**V**	305	311	KFK**V**VKE	0.988
309	**V**	306	312	FKV**V**KEI	0.984
310	**K**	307	313	KVV**K**EIA	0.991
311	**E**	308	314	VVK**E**IAE	0.986
312	**I**	309	315	VKE**I**AET	0.984

313	**A**	310	316	KEI**A**ETQ	0.994
314	**E**	311	317	EIA**E**TQH	1.004
315	**T**	312	318	IAE**T**QHG	1.017
316	**Q**	313	319	AET**Q**HGT	1.027
317	**H**	314	320	ETQ**H**GTI	1.020
318	**G**	315	321	TQH**G**TIV	1.006
319	**T**	316	322	QHG**T**IVV	0.984
320	**I**	317	323	HGT**I**VVR	0.955
321	**V**	318	324	GTI**V**VRV	0.940
322	**V**	319	325	TIV**V**RVQ	0.927
323	**R**	320	326	IVV**R**VQY	0.924
324	**V**	321	327	VVR**V**QYE	0.938
325	**Q**	322	328	VRV**Q**YEG	0.957
326	**Y**	323	329	RVQ**Y**EGD	0.987
327	**E**	324	330	VQY**E**GDG	1.026
328	**G**	325	331	QYE**G**DGS	1.065
329	**D**	326	332	YEG**D**GSP	1.090
330	**G**	327	333	EGD**G**SPC	1.102
331	**S**	328	334	GDG**S**PCK	1.096
332	**P**	329	335	DGS**P**CKI	1.072
333	**C**	330	336	GSP**C**KIP	1.043
334	**K**	331	337	SPC**K**IPF	1.015
335	**I**	332	338	PCK**I**PFE	0.986
336	**P**	333	339	CKI**P**FEI	0.960
337	**F**	334	340	KIP**F**EIM	0.939
338	**E**	335	341	IPF**E**IMD	0.920
339	**I**	336	342	PFE**I**MDL	0.911
340	**M**	337	343	FEI**M**DLE	0.918
341	**D**	338	344	EIM**D**LEK	0.944
342	**L**	339	345	IMD**L**EKR	0.975
343	**E**	340	346	MDL**E**KRH	1.004
344	**K**	341	347	DLE**K**RHV	1.015
345	**R**	342	348	LEK**R**HVL	1.005
346	**H**	343	349	EKR**H**VLG	0.989
347	**V**	344	350	KRH**V**LGR	0.978
348	**L**	345	351	RHV**L**GRL	0.979

349	**G**	346	352	HVL**G**RLI	0.983
350	**R**	347	353	VLG**R**LIT	0.980
351	**L**	348	354	LGR**L**ITV	0.969
352	**I**	349	355	GRL**I**TVN	0.958
353	**T**	350	356	RLI**T**VNP	0.963
354	**V**	351	357	LIT**V**NPI	0.972
355	**N**	352	358	ITV**N**PIV	0.981
356	**P**	353	359	TVN**P**IVT	0.990
357	**I**	354	360	VNP**I**VTE	0.987
358	**V**	355	361	NPI**V**TEK	1.000
359	**T**	356	362	PIV**T**EKD	1.022
360	**E**	357	363	IVT**E**KDS	1.049
361	**K**	358	364	VTE**K**DSP	1.075
362	**D**	359	365	TEK**D**SPV	1.082
363	**S**	360	366	EKD**S**PVN	1.075
364	**P**	361	367	KDS**P**VNI	1.051
365	**V**	362	368	DSP**V**NIE	1.018
366	**N**	363	369	SPV**N**IEA	0.995
367	**I**	364	370	PVN**I**EAE	0.985
368	**E**	365	371	VNI**E**AEP	0.989
369	**A**	366	372	NIE**A**EPP	1.012
370	**E**	367	373	IEA**E**PPF	1.025
371	**P**	368	374	EAE**P**PFG	1.031
372	**P**	369	375	AEP**P**FGD	1.033
373	**F**	370	376	EPP**F**GDS	1.023
374	**G**	371	377	PPF**G**DSY	1.019
375	**D**	372	378	PFG**D**SYI	1.005
376	**S**	373	379	FGD**S**YII	0.982
377	**Y**	374	380	GDS**Y**III	0.951
378	**I**	375	381	DSY**I**IIG	0.921
379	**I**	376	382	SYI**I**IGV	0.907
380	**I**	377	383	YII**I**GVE	0.914
381	**G**	378	384	III**G**VEP	0.941
382	**V**	379	385	IIG**V**EPG	0.982
383	**E**	380	386	IGV**E**PGQ	1.019
384	**P**	381	387	GVE**P**GQL	1.041

385	**G**	382	388	VEP**G**QLK	1.054
386	**Q**	383	389	EPG**Q**LKL	1.042
387	**L**	384	390	PGQ**L**KLS	1.020
388	**K**	385	391	GQL**K**LSW	0.999
389	**L**	386	392	QLK**L**SWF	0.970
390	**S**	387	393	LKL**S**WFK	0.958
391	**W**	388	394	KLS**W**FKK	0.964
392	**F**	389	395	LSW**F**KKG	0.987
393	**K**	390	396	SWF**K**KGS	1.035
394	**K**	391	397	WFK**K**GSS	1.073
395	**G**	392	398	FKK**G**SSI	1.096
396	**S**	393	399	KKG**S**SIG	1.098
397	**S**	394	400	KGS**S**IGQ	1.075
398	**I**	395	401	GSS**I**GQM	1.042
399	**G**	396	402	SSI**G**QMF	1.010
400	**Q**	397	403	SIG**Q**MFE	0.984
401	**M**	398	404	IGQ**M**FET	0.969
402	**F**	399	405	GQM**F**ETT	0.977
403	**E**	400	406	QMF**E**TTM	0.994
404	**T**	401	407	MFE**T**TMR	1.010
405	**T**	402	408	FET**T**MRG	1.022
406	**M**	403	409	ETT**M**RGA	1.021
407	**R**	404	410	TTM**R**GAK	1.026
408	**G**	405	411	TMR**G**AKR	1.032
409	**A**	406	412	MRG**A**KRM	1.029
410	**K**	407	413	RGA**K**RMA	1.016
411	**R**	408	414	GAK**R**MAI	0.983
412	**M**	409	415	AKR**M**AIL	0.945
413	**A**	410	416	KRM**A**ILG	0.930
414	**I**	411	417	RMA**I**LGD	0.932
415	**L**	412	418	MAI**L**GDT	0.959
416	**G**	413	419	AIL**G**DTA	0.990
417	**D**	414	420	ILG**D**TAW	1.001
418	**T**	415	421	LGD**T**AWD	0.997
419	**A**	416	422	GDT**A**WDF	0.974
420	**W**	417	423	DTA**W**DFG	0.959

421	**D**	418	424	TAW**D**FGS	0.959
422	**F**	419	425	AWD**F**GSL	0.968
423	**G**	420	426	WDF**G**SLG	0.991
424	**S**	421	427	DFG**S**LGG	1.009
425	**L**	422	428	FGS**L**GGV	1.010
426	**G**	423	429	GSL**G**GVF	1.006
427	**G**	424	430	SLG**G**VFT	0.996
428	**V**	425	431	LGG**V**FTS	0.986
429	**F**	426	432	GGV**F**TSI	0.988
430	**T**	427	433	GVF**T**SIG	1.002
431	**S**	428	434	VFT**S**IGK	1.017
432	**I**	429	435	FTS**I**GKA	1.025
433	**G**	430	436	TSI**G**KAL	1.024
434	**K**	431	437	SIG**K**ALH	1.009
435	**A**	432	438	IGK**A**LHQ	0.979
436	**L**	433	439	GKA**L**HQV	0.955
437	**H**	434	440	KAL**H**QVF	0.935
438	**Q**	435	441	ALH**Q**VFG	0.920
439	**V**	436	442	LHQ**V**FGA	0.920
440	**F**	437	443	HQV**F**GAI	0.918
441	**G**	438	444	QVF**G**AIY	0.917
442	**A**	439	445	VFG**A**IYG	0.919
443	**I**	440	446	FGA**I**YGA	0.917
444	**Y**	441	447	GAI**Y**GAA	0.919
445	**G**	442	448	AIY**G**AAF	0.919
446	**A**	443	449	IYG**A**AFS	0.927
447	**A**	444	450	YGA**A**FSG	0.941
448	**F**	445	451	GAA**F**SGV	0.959
449	**S**	446	452	AAF**S**GVS	0.979
450	**G**	447	453	AFS**G**VSW	0.984
451	**V**	448	454	FSG**V**SWT	0.976
452	**S**	449	455	SGV**S**WTM	0.960
453	**W**	450	456	GVS**W**TMK	0.952
454	**T**	451	457	VSW**T**MKI	0.949
455	**M**	452	458	SWT**M**KIL	0.952
456	**K**	453	459	WTM**K**ILI	0.954

457	**I**	454	460	TMK**I**LIG	0.940
458	**L**	455	461	MKI**L**IGV	0.929
459	**I**	456	462	KIL**I**GVV	0.919
460	**G**	457	463	ILI**G**VVI	0.913
461	**V**	458	464	LIG**V**VIT	0.915
462	**V**	459	465	IGV**V**ITW	0.914
463	**I**	460	466	GVV**I**TWI	0.913
464	**T**	461	467	VVI**T**WIG	0.914
465	**W**	462	468	VIT**W**IGM	0.915
466	**I**	463	469	ITW**I**GMN	0.922
467	**G**	464	470	TWI**G**MNS	0.947
468	**M**	465	471	WIG**M**NSR	0.978
469	**N**	466	472	IGM**N**SRS	1.022
470	**S**	467	473	GMN**S**RST	1.065
471	**R**	468	474	MNS**R**STS	1.082
472	**S**	469	475	NSR**S**TSL	1.088
473	**T**	470	476	SRS**T**SLS	1.065
474	**S**	471	477	RST**S**LSV	1.032
475	**L**	472	478	STS**L**SVS	0.999
476	**S**	473	479	TSL**S**VSL	0.970
477	**V**	474	480	SLS**V**SLV	0.955
478	**S**	475	481	LSV**S**LVL	0.943
479	**L**	476	482	SVS**L**VLV	0.938
480	**V**	477	483	VSL**V**LVG	0.931
481	**L**	478	484	SLV**L**VGV	0.926
482	**V**	479	485	LVL**V**GVV	0.926
483	**G**	480	486	VLV**G**VVT	0.925
484	**V**	481	487	LVG**V**VTL	0.929
485	**V**	482	488	VGV**V**TLY	0.926
486	**T**	483	489	GVV**T**LYL	0.926
487	**L**	484	490	VVT**L**YLG	0.924
488	**Y**	485	491	VTL**Y**LGV	0.918
489	**L**	486	492	TLY**L**GVM	0.915
490	**G**	487	493	LYL**G**VMV	0.908
491	**V**	488	494	YLG**V**MVQ	0.899
