# Supplementary material for: Computational Prediction and Analysis of Envelop Glycoprotein Epitopes of DENV-2 and DENV-3 Pakistani Isolates: A First Step towards Dengue Vaccine Development
Source: PLoS One. 2015 Mar 16;10(3):e0119854. doi: 10.1371/journal.pone.0119854 (PMC4361635; doi:10.1371/journal.pone.0119854)
Supplement: S4 Table — (PDF) [file pone.0119854.s006.pdf]

IEDB Analysis Resource

Antibody Epitope Prediction | Example Sequences | Tutorial | External Links
Reference | Download | Contact

Karplus & Schulz Flexibility Prediction Result Data Table

Average: 0.997 **Minimum:** 0.892 **Maximum:** 1.122 Download data to file

Position ▲ ▼	Residue	Peptide start position	Peptide end position	Peptide	Score ▲ ▼
4	**V**	1	7	MRC**V**GVG	0.929
5	**G**	2	8	RCV**G**VGN	0.955
6	**V**	3	9	CVG**V**GNR	0.989
7	**G**	4	10	VGV**G**NRD	1.025
8	**N**	5	11	GVG**N**RDF	1.043
9	**R**	6	12	VGN**R**DFV	1.037
10	**D**	7	13	GNR**D**FVE	1.023
11	**F**	8	14	NRD**F**VEG	1.000
12	**V**	9	15	RDF**V**EGL	0.989
13	**E**	10	16	DFV**E**GLS	0.997
14	**G**	11	17	FVE**G**LSG	1.005
15	**L**	12	18	VEG**L**SGA	1.016
16	**S**	13	19	EGL**S**GAT	1.022
17	**G**	14	20	GLS**G**ATW	1.012
18	**A**	15	21	LSG**A**TWV	0.995
19	**T**	16	22	SGA**T**WVD	0.970
20	**W**	17	23	GAT**W**VDV	0.948
21	**V**	18	24	ATW**V**DVV	0.933
22	**D**	19	25	TWV**D**VVL	0.928
23	**V**	20	26	WVD**V**VLE	0.929
24	**V**	21	27	VDV**V**LEH	0.935
25	**L**	22	28	DVV**L**EHG	0.948

26	**E**	23	29	VVL**E**HGG	0.965
27	**H**	24	30	VLE**H**GGC	0.978
28	**G**	25	31	LEH**G**GCV	0.984
29	**G**	26	32	EHG**G**CVT	0.983
30	**C**	27	33	HGG**C**VTT	0.977
31	**V**	28	34	GGC**V**TTM	0.974
32	**T**	29	35	GCV**T**TMA	0.975
33	**T**	30	36	CVT**T**MAK	0.978
34	**M**	31	37	VTT**M**AKN	0.984
35	**A**	32	38	TTM**A**KNK	1.006
36	**K**	33	39	TMA**K**NKP	1.033
37	**N**	34	40	MAK**N**KPT	1.057
38	**K**	35	41	AKN**K**PTL	1.067
39	**P**	36	42	KNK**P**TLD	1.052
40	**T**	37	43	NKP**T**LDI	1.027
41	**L**	38	44	KPT**L**DIE	0.997
42	**D**	39	45	PTL**D**IEL	0.977
43	**I**	40	46	TLD**I**ELQ	0.972
44	**E**	41	47	LDI**E**LQK	0.976
45	**L**	42	48	DIE**L**QKT	0.997
46	**Q**	43	49	IEL**Q**KTE	1.021
47	**K**	44	50	ELQ**K**TEA	1.041
48	**T**	45	51	LQK**T**EAT	1.052
49	**E**	46	52	QKT**E**ATQ	1.052
50	**A**	47	53	KTE**A**TQL	1.043
51	**T**	48	54	TEA**T**QLA	1.026
52	**Q**	49	55	EAT**Q**LAT	1.006
53	**L**	50	56	ATQ**L**ATL	0.984
54	**A**	51	57	TQL**A**TLR	0.971
55	**T**	52	58	QLA**T**LRK	0.973
56	**L**	53	59	LAT**L**RKL	0.983
57	**R**	54	60	ATL**R**KLC	0.995
58	**K**	55	61	TLR**K**LCI	0.994
59	**L**	56	62	LRK**L**CIE	0.982
60	**C**	57	63	RKL**C**IEG	0.979
61	**I**	58	64	KLC**I**EGK	0.989

62	**E**	59	65	LCI**E**GKI	1.014
63	**G**	60	66	CIE**G**KIT	1.043
64	**K**	61	67	IEG**K**ITN	1.052
65	**I**	62	68	EGK**I**TNI	1.046
66	**T**	63	69	GKI**T**NIT	1.037
67	**N**	64	70	KIT**N**ITT	1.028
68	**I**	65	71	ITN**I**TTD	1.029
69	**T**	66	72	TNI**T**TDS	1.045
70	**T**	67	73	NIT**T**DSR	1.057
71	**D**	68	74	ITT**D**SRC	1.061
72	**S**	69	75	TTD**S**RCP	1.066
73	**R**	70	76	TDS**R**CPT	1.055
74	**C**	71	77	DSR**C**PTQ	1.054
75	**P**	72	78	SRC**P**TQG	1.071
76	**T**	73	79	RCP**T**QGE	1.082
77	**Q**	74	80	CPT**Q**GEA	1.091
78	**G**	75	81	PTQ**G**EAV	1.075
79	**E**	76	82	TQG**E**AVL	1.037
80	**A**	77	83	QGE**A**VLP	1.005
81	**V**	78	84	GEA**V**LPE	0.991
82	**L**	79	85	EAV**L**PEE	1.003
83	**P**	80	86	AVL**P**EEQ	1.039
84	**E**	81	87	VLP**E**EQD	1.069
85	**E**	82	88	LPE**E**QDQ	1.095
86	**Q**	83	89	PEE**Q**DQN	1.103
87	**D**	84	90	EEQ**D**QNY	1.084
88	**Q**	85	91	EQD**Q**NYV	1.061
89	**N**	86	92	QDQ**N**YVC	1.015
90	**Y**	87	93	DQN**Y**VCK	0.978
91	**V**	88	94	QNY**V**CKH	0.959
92	**C**	89	95	NYV**C**KHT	0.949
93	**K**	90	96	YVC**K**HTY	0.960
94	**H**	91	97	VCK**H**TYV	0.960
95	**T**	92	98	CKH**T**YVD	0.963
96	**Y**	93	99	KHT**Y**VDR	0.971
97	**V**	94	100	HTY**V**DRG	0.983

98	**D**	95	101	TYV**D**RGW	1.003
99	**R**	96	102	YVD**R**GWG	1.014
100	**G**	97	103	VDR**G**WGN	1.023
101	**W**	98	104	DRG**W**GNG	1.025
102	**G**	99	105	RGW**G**NGC	1.029
103	**N**	100	106	GWG**N**GCG	1.028
104	**G**	101	107	WGN**G**CGL	1.013
105	**C**	102	108	GNG**C**GLF	0.990
106	**G**	103	109	NGC**G**LFG	0.970
107	**L**	104	110	GCG**L**FGK	0.968
108	**F**	105	111	CGL**F**GKG	0.987
109	**G**	106	112	GLF**G**KGS	1.022
110	**K**	107	113	LFG**K**GSL	1.048
111	**G**	108	114	FGK**G**SLV	1.052
112	**S**	109	115	GKG**S**LVT	1.032
113	**L**	110	116	KGS**L**VTC	0.993
114	**V**	111	117	GSL**V**TCA	0.958
115	**T**	112	118	SLV**T**CAK	0.942
116	**C**	113	119	LVT**C**AKF	0.938
117	**A**	114	120	VTC**A**KFQ	0.946
118	**K**	115	121	TCA**K**FQC	0.950
119	**F**	116	122	CAK**F**QCL	0.940
120	**Q**	117	123	AKF**Q**CLE	0.936
121	**C**	118	124	KFQ**C**LEP	0.945
122	**L**	119	125	FQC**L**EPI	0.966
123	**E**	120	126	QCL**E**PIE	0.998
124	**P**	121	127	CLE**P**IEG	1.029
125	**I**	122	128	LEP**I**EGK	1.048
126	**E**	123	129	EPI**E**GKV	1.055
127	**G**	124	130	PIE**G**KVV	1.050
128	**K**	125	131	IEG**K**VVQ	1.022
129	**V**	126	132	EGK**V**VQY	0.987
130	**V**	127	133	GKV**V**QYE	0.963
131	**Q**	128	134	KVV**Q**YEN	0.951
132	**Y**	129	135	VVQ**Y**ENL	0.960
133	**E**	130	136	VQY**E**NLK	0.983

134	**N**	131	137	QYE**N**LKY	0.995
135	**L**	132	138	YEN**L**KYT	0.997
136	**K**	133	139	ENL**K**YTV	0.991
137	**Y**	134	140	NLK**Y**TVI	0.968
138	**T**	135	141	LKY**T**VII	0.945
139	**V**	136	142	KYT**V**IIT	0.926
140	**I**	137	143	YTV**I**ITV	0.911
141	**I**	138	144	TVI**I**TVH	0.911
142	**T**	139	145	VII**T**VHT	0.926
143	**V**	140	146	IIT**V**HTG	0.954
144	**H**	141	147	ITV**H**TGD	0.989
145	**T**	142	148	TVH**T**GDQ	1.023
146	**G**	143	149	VHT**G**DQH	1.043
147	**D**	144	150	HTG**D**QHQ	1.034
148	**Q**	145	151	TGD**Q**HQV	1.015
149	**H**	146	152	GDQ**H**QVG	0.993
150	**Q**	147	153	DQH**Q**VGN	0.983
151	**V**	148	154	QHQ**V**GNE	1.002
152	**G**	149	155	HQV**G**NET	1.030
153	**N**	150	156	QVG**N**ETQ	1.065
154	**E**	151	157	VGN**E**TQG	1.086
155	**T**	152	158	GNE**T**QGV	1.088
156	**Q**	153	159	NET**Q**GVT	1.076
157	**G**	154	160	ETQ**G**VTA	1.047
158	**V**	155	161	TQG**V**TAE	1.017
159	**T**	156	162	QGV**T**AEI	0.995
160	**A**	157	163	GVT**A**EIT	0.989
161	**E**	158	164	VTA**E**ITP	0.992
162	**I**	159	165	TAE**I**TPQ	1.007
163	**T**	160	166	AEI**T**PQA	1.024
164	**P**	161	167	EIT**P**QAS	1.033
165	**Q**	162	168	ITP**Q**AST	1.042
166	**A**	163	169	TPQ**A**STT	1.048
167	**S**	164	170	PQA**S**TTE	1.052
168	**T**	165	171	QAS**T**TEA	1.049
169	**T**	166	172	AST**T**EAI	1.032

170	**E**	167	173	STT**E**AIL	1.006
171	**A**	168	174	TTE**A**ILP	0.983
172	**I**	169	175	TEA**I**LPE	0.972
173	**L**	170	176	EAI**L**PEY	0.980
174	**P**	171	177	AIL**P**EYG	0.999
175	**E**	172	178	ILP**E**YGT	1.013
176	**Y**	173	179	LPE**Y**GTL	1.017
177	**G**	174	180	PEY**G**TLG	1.014
178	**T**	175	181	EYG**T**LGL	1.000
179	**L**	176	182	YGT**L**GLE	0.981
180	**G**	177	183	GTL**G**LEC	0.966
181	**L**	178	184	TLG**L**ECS	0.962
182	**E**	179	185	LGL**E**CSP	0.967
183	**C**	180	186	GLE**C**SPR	0.987
184	**S**	181	187	LEC**S**PRT	1.014
185	**P**	182	188	ECS**P**RTG	1.032
186	**R**	183	189	CSP**R**TGL	1.040
187	**T**	184	190	SPR**T**GLD	1.035
188	**G**	185	191	PRT**G**LDF	1.013
189	**L**	186	192	RTG**L**DFN	0.990
190	**D**	187	193	TGL**D**FNE	0.976
191	**F**	188	194	GLD**F**NEM	0.965
192	**N**	189	195	LDF**N**EMI	0.959
193	**E**	190	196	DFN**E**MIL	0.950
194	**M**	191	197	FNE**M**ILL	0.932
195	**I**	192	198	NEM**I**LLT	0.923
196	**L**	193	199	EMI**L**LTM	0.922
197	**L**	194	200	MIL**L**TMK	0.937
198	**T**	195	201	ILL**T**MKN	0.966
199	**M**	196	202	LLT**M**KNK	1.000
200	**K**	197	203	LTM**K**NKA	1.029
201	**N**	198	204	TMK**N**KAW	1.030
202	**K**	199	205	MKN**K**AWM	1.002
203	**A**	200	206	KNK**A**WMV	0.955
204	**W**	201	207	NKA**W**MVH	0.908
205	**M**	202	208	KAW**M**VHR	0.892 (minimum)

206	**V**	203	209	AWM**V**HRQ	0.910
207	**H**	204	210	WMV**H**RQW	0.938
208	**R**	205	211	MVH**R**QWF	0.964
209	**Q**	206	212	VHR**Q**WFF	0.969
210	**W**	207	213	HRQ**W**FFD	0.954
211	**F**	208	214	RQW**F**FDL	0.941
212	**F**	209	215	QWF**F**DLP	0.932
213	**D**	210	216	WFF**D**LPL	0.934
214	**L**	211	217	FFD**L**PLP	0.942
215	**P**	212	218	FDL**P**LPW	0.943
216	**L**	213	219	DLP**L**PWT	0.952
217	**P**	214	220	LPL**P**WTS	0.969
218	**W**	215	221	PLP**W**TSG	0.993
219	**T**	216	222	LPW**T**SGA	1.028
220	**S**	217	223	PWT**S**GAT	1.054
221	**G**	218	224	WTS**G**ATT	1.062
222	**A**	219	225	TSG**A**TTE	1.067
223	**T**	220	226	SGA**T**TET	1.066
224	**T**	221	227	GAT**T**ETP	1.066
225	**E**	222	228	ATT**E**TPT	1.070
226	**T**	223	229	TTE**T**PTW	1.060
227	**P**	224	230	TET**P**TWN	1.044
228	**T**	225	231	ETP**T**WNR	1.027
229	**W**	226	232	TPT**W**NRK	1.014
230	**N**	227	233	PTW**N**RKE	1.018
231	**R**	228	234	TWN**R**KEL	1.024
232	**K**	229	235	WNR**K**ELL	1.023
233	**E**	230	236	NRK**E**LLV	1.010
234	**L**	231	237	RKE**L**LVT	0.983
235	**L**	232	238	KEL**L**VTF	0.957
236	**V**	233	239	ELL**V**TFK	0.948
237	**T**	234	240	LLV**T**FKN	0.955
238	**F**	235	241	LVT**F**KNA	0.969
239	**K**	236	242	VTF**K**NAH	0.982
240	**N**	237	243	TFK**N**AHA	0.977
241	**A**	238	244	FKN**A**HAK	0.972

242	**H**	239	245	KNA**H**AKK	0.975
243	**A**	240	246	NAH**A**KKQ	0.998
244	**K**	241	247	AHA**K**KQE	1.034
245	**K**	242	248	HAK**K**QEV	1.056
246	**Q**	243	249	AKK**Q**EVV	1.058
247	**E**	244	250	KKQ**E**VVV	1.027
248	**V**	245	251	KQE**V**VVL	0.985
249	**V**	246	252	QEV**V**VLG	0.961
250	**V**	247	253	EVV**V**LGS	0.964
251	**L**	248	254	VVV**L**GSQ	0.998
252	**G**	249	255	VVL**G**SQE	1.045
253	**S**	250	256	VLG**S**QEG	1.085
254	**Q**	251	257	LGS**Q**EGA	1.095
255	**E**	252	258	GSQ**E**GAM	1.067
256	**G**	253	259	SQE**G**AMH	1.022
257	**A**	254	260	QEG**A**MHT	0.970
258	**M**	255	261	EGA**M**HTA	0.931
259	**H**	256	262	GAM**H**TAL	0.926
260	**T**	257	263	AMH**T**ALT	0.935
261	**A**	258	264	MHT**A**LTG	0.958
262	**L**	259	265	HTA**L**TGA	0.986
263	**T**	260	266	TAL**T**GAT	1.011
264	**G**	261	267	ALT**G**ATE	1.028
265	**A**	262	268	LTG**A**TEI	1.036
266	**T**	263	269	TGA**T**EIQ	1.038
267	**E**	264	270	GAT**E**IQN	1.038
268	**I**	265	271	ATE**I**QNS	1.047
269	**Q**	266	272	TEI**Q**NSG	1.067
270	**N**	267	273	EIQ**N**SGG	1.095
271	**S**	268	274	IQN**S**GGT	1.117
272	**G**	269	275	QNS**G**GTS	1.122<br>(maximum)
273	**G**	270	276	NSG**G**TSI	1.104
274	**T**	271	277	SGG**T**SIF	1.063
275	**S**	272	278	GGT**S**IFA	1.018
276	**I**	273	279	GTS**I**FAG	0.972
277	**F**	274	280	TSI**F**AGH	0.946

278	**A**	275	281	SIF**A**GHL	0.937
279	**G**	276	282	IFA**G**HLK	0.943
280	**H**	277	283	FAG**H**LKC	0.959
281	**L**	278	284	AGH**L**KCR	0.968
282	**K**	279	285	GHL**K**CRL	0.974
283	**C**	280	286	HLK**C**RLK	0.974
284	**R**	281	287	LKC**R**LKM	0.970
285	**L**	282	288	KCR**L**KMD	0.981
286	**K**	283	289	CRL**K**MDK	1.000
287	**M**	284	290	RLK**M**DKL	1.012
288	**D**	285	291	LKM**D**KLE	1.024
289	**K**	286	292	KMD**K**LEL	1.017
290	**L**	287	293	MDK**L**ELK	1.003
291	**E**	288	294	DKL**E**LKG	1.000
292	**L**	289	295	KLE**L**KGM	0.997
293	**K**	290	296	LEL**K**GMS	1.000
294	**G**	291	297	ELK**G**MSY	0.994
295	**M**	292	298	LKG**M**SYA	0.973
296	**S**	293	299	KGM**S**YAM	0.942
297	**Y**	294	300	GMS**Y**AMC	0.912
298	**A**	295	301	MSY**A**MCT	0.898
299	**M**	296	302	SYA**M**CTN	0.908
300	**C**	297	303	YAM**C**TNT	0.942
301	**T**	298	304	AMC**T**NTF	0.981
302	**N**	299	305	MCT**N**TFV	1.004
303	**T**	300	306	CTN**T**FVL	1.004
304	**F**	301	307	TNT**F**VLK	0.989
305	**V**	302	308	NTF**V**LKK	0.984
306	**L**	303	309	TFV**L**KKE	0.995
307	**K**	304	310	FVL**K**KEV	1.018
308	**K**	305	311	VLK**K**EVS	1.036
309	**E**	306	312	LKK**E**VSE	1.043
310	**V**	307	313	KKE**V**SET	1.044
311	**S**	308	314	KEV**S**ETQ	1.047
312	**E**	309	315	EVS**E**TQH	1.049
313	**T**	310	316	VSE**T**QHG	1.046

314	**Q**	311	317	SET**Q**HGT	1.040
315	**H**	312	318	ETQ**H**GTI	1.023
316	**G**	313	319	TQH**G**TIL	1.006
317	**T**	314	320	QHG**T**ILI	0.983
318	**I**	315	321	HGT**I**LIK	0.962
319	**L**	316	322	GTI**L**IKV	0.955
320	**I**	317	323	TIL**I**KVE	0.952
321	**K**	318	324	ILI**K**VEY	0.963
322	**V**	319	325	LIK**V**EYK	0.975
323	**E**	320	326	IKV**E**YKG	0.992
324	**Y**	321	327	KVE**Y**KGE	1.018
325	**K**	322	328	VEY**K**GED	1.048
326	**G**	323	329	EYK**G**EDA	1.073
327	**E**	324	330	YKG**E**DAP	1.076
328	**D**	325	331	KGE**D**APC	1.059
329	**A**	326	332	GED**A**PCK	1.033
330	**P**	327	333	EDA**P**CKI	1.007
331	**C**	328	334	DAP**C**KIP	0.995
332	**K**	329	335	APC**K**IPF	0.988
333	**I**	330	336	PCK**I**PFS	0.983
334	**P**	331	337	CKI**P**FST	0.985
335	**F**	332	338	KIP**F**STE	0.998
336	**S**	333	339	IPF**S**TED	1.018
337	**T**	334	340	PFS**T**EDG	1.045
338	**E**	335	341	FST**E**DGQ	1.073
339	**D**	336	342	STE**D**GQG	1.094
340	**G**	337	343	TED**G**QGK	1.112
341	**Q**	338	344	EDG**Q**GKA	1.111
342	**G**	339	345	DGQ**G**KAH	1.092
343	**K**	340	346	GQG**K**AHN	1.061
344	**A**	341	347	QGK**A**HNG	1.031
345	**H**	342	348	GKA**H**NGR	1.018
346	**N**	343	349	KAH**N**GRL	1.019
347	**G**	344	350	AHN**G**RLI	1.021
348	**R**	345	351	HNG**R**LIT	1.006
349	**L**	346	352	NGR**L**ITA	0.988

350	**I**	347	353	GRL**I**TAN	0.971
351	**T**	348	354	RLI**T**ANP	0.974
352	**A**	349	355	LIT**A**NPV	0.989
353	**N**	350	356	ITA**N**PVV	0.996
354	**P**	351	357	TAN**P**VVT	1.004
355	**V**	352	358	ANP**V**VTK	1.000
356	**V**	353	359	NPV**V**TKK	1.006
357	**T**	354	360	PVV**T**KKE	1.030
358	**K**	355	361	VVT**K**KEE	1.054
359	**K**	356	362	VTK**K**EEP	1.077
360	**E**	357	363	TKK**E**EPV	1.083
361	**E**	358	364	KKE**E**PVN	1.068
362	**P**	359	365	KEE**P**VNI	1.044
363	**V**	360	366	EEP**V**NIE	1.012
364	**N**	361	367	EPV**N**IEA	0.990
365	**I**	362	368	PVN**I**EAE	0.985
366	**E**	363	369	VNI**E**AEP	0.989
367	**A**	364	370	NIE**A**EPP	1.012
368	**E**	365	371	IEA**E**PPF	1.025
369	**P**	366	372	EAE**P**PFG	1.031
370	**P**	367	373	AEP**P**FGE	1.037
371	**F**	368	374	EPP**F**GES	1.038
372	**G**	369	375	PPF**G**ESN	1.051
373	**E**	370	376	PFG**E**SNI	1.054
374	**S**	371	377	FGE**S**NIV	1.043
375	**N**	372	378	GES**N**IVI	1.010
376	**I**	373	379	ESN**I**VIG	0.967
377	**V**	374	380	SNI**V**IGI	0.940
378	**I**	375	381	NIV**I**GIG	0.932
379	**G**	376	382	IVI**G**IGD	0.951
380	**I**	377	383	VIG**I**GDN	0.980
381	**G**	378	384	IGI**G**DNA	0.998
382	**D**	379	385	GIG**D**NAL	1.003
383	**N**	380	386	IGD**N**ALK	1.002
384	**A**	381	387	GDN**A**LKI	0.994
385	**L**	382	388	DNA**L**KIN	0.989

386	**K**	383	389	NAL**K**INW	0.987
387	**I**	384	390	ALK**I**NWY	0.975
388	**N**	385	391	LKI**N**WYK	0.968
389	**W**	386	392	KIN**W**YKK	0.973
390	**Y**	387	393	INW**Y**KKG	0.994
391	**K**	388	394	NWY**K**KGS	1.038
392	**K**	389	395	WYK**K**GSS	1.075
393	**G**	390	396	YKK**G**SSI	1.097
394	**S**	391	397	KKG**S**SIG	1.098
395	**S**	392	398	KGS**S**IGK	1.078
396	**I**	393	399	GSS**I**GKM	1.049
397	**G**	394	400	SSI**G**KMF	1.020
398	**K**	395	401	SIG**K**MFE	0.991
399	**M**	396	402	IGK**M**FEA	0.964
400	**F**	397	403	GKM**F**EAT	0.953
401	**E**	398	404	KMF**E**ATA	0.957
402	**A**	399	405	MFE**A**TAR	0.973
403	**T**	400	406	FEA**T**ARG	0.991
404	**A**	401	407	EAT**A**RGA	1.012
405	**R**	402	408	ATA**R**GAR	1.024
406	**G**	403	409	TAR**G**ARR	1.030
407	**A**	404	410	ARG**A**RRM	1.024
408	**R**	405	411	RGA**R**RMA	1.003
409	**R**	406	412	GAR**R**MAI	0.973
410	**M**	407	413	ARR**M**AIL	0.938
411	**A**	408	414	RRM**A**ILG	0.926
412	**I**	409	415	RMA**I**LGD	0.932
413	**L**	410	416	MAI**L**GDT	0.959
414	**G**	411	417	AIL**G**DTA	0.990
415	**D**	412	418	ILG**D**TAW	1.001
416	**T**	413	419	LGD**T**AWD	0.997
417	**A**	414	420	GDT**A**WDF	0.974
418	**W**	415	421	DTA**W**DFG	0.959
419	**D**	416	422	TAW**D**FGS	0.959
420	**F**	417	423	AWD**F**GSV	0.969
421	**G**	418	424	WDF**G**SVG	0.993

422	**S**	419	425	DFG**S**VGG	1.011
423	**V**	420	426	FGS**V**GGV	1.014
424	**G**	421	427	GSV**G**GVL	1.012
425	**G**	422	428	SVG**G**VLN	1.001
426	**V**	423	429	VGG**V**LNS	0.990
427	**L**	424	430	GGV**L**NSL	0.989
428	**N**	425	431	GVL**N**SLG	0.995
429	**S**	426	432	VLN**S**LGK	1.008
430	**L**	427	433	LNS**L**GKM	1.009
431	**G**	428	434	NSL**G**KMV	1.003
432	**K**	429	435	SLG**K**MVH	0.988
433	**M**	430	436	LGK**M**VHQ	0.955
434	**V**	431	437	GKM**V**HQI	0.935
435	**H**	432	438	KMV**H**QIF	0.919
436	**Q**	433	439	MVH**Q**IFG	0.914
437	**I**	434	440	VHQ**I**FGS	0.932
438	**F**	435	441	HQI**F**GSA	0.951
439	**G**	436	442	QIF**G**SAY	0.971
440	**S**	437	443	IFG**S**AYT	0.979
441	**A**	438	444	FGS**A**YTA	0.970
442	**Y**	439	445	GSA**Y**TAL	0.957
443	**T**	440	446	SAY**T**ALF	0.941
444	**A**	441	447	AYT**A**LFS	0.939
445	**L**	442	448	YTA**L**FSG	0.950
446	**F**	443	449	TAL**F**SGV	0.965
447	**S**	444	450	ALF**S**GVS	0.983
448	**G**	445	451	LFS**G**VSW	0.985
449	**V**	446	452	FSG**V**SWV	0.974
450	**S**	447	453	SGV**S**WVM	0.951
451	**W**	448	454	GVS**W**VMK	0.935
452	**V**	449	455	VSW**V**MKI	0.933
453	**M**	450	456	SWV**M**KIG	0.940
454	**K**	451	457	WVM**K**IGI	0.963
455	**I**	452	458	VMK**I**GIG	0.969
456	**G**	453	459	MKI**G**IGV	0.966
457	**I**	454	460	KIG**I**GVL	0.961

458	**G**	455	461	IGI**G**VLL	0.946
459	**V**	456	462	GIG**V**LLT	0.939
460	**L**	457	463	IGV**L**LTW	0.936
461	**L**	458	464	GVL**L**TWI	0.932
462	**T**	459	465	VLL**T**WIG	0.928
463	**W**	460	466	LLT**W**IGL	0.925
464	**I**	461	467	LTW**I**GLN	0.929
465	**G**	462	468	TWI**G**LNS	0.951
466	**L**	463	469	WIG**L**NSK	0.987
467	**N**	464	470	IGL**N**SKN	1.030
468	**S**	465	471	GLN**S**KNT	1.071
469	**K**	466	472	LNS**K**NTS	1.088
470	**N**	467	473	NSK**N**TSM	1.085
471	**T**	468	474	SKN**T**SMS	1.060
472	**S**	469	475	KNT**S**MSF	1.022
473	**M**	470	476	NTS**M**SFS	0.984
474	**S**	471	477	TSM**S**FSC	0.951
475	**F**	472	478	SMS**F**SCI	0.927
476	**S**	473	479	MSF**S**CIA	0.909
477	**C**	474	480	SFS**C**IAI	0.897
478	**I**	475	481	FSC**I**AIG	0.893
479	**A**	476	482	SCI**A**IGI	0.892
480	**I**	477	483	CIA**I**GII	0.894
481	**G**	478	484	IAI**G**IIT	0.901
482	**I**	479	485	AIG**I**ITL	0.907
483	**I**	480	486	IGI**I**TLY	0.909
484	**T**	481	487	GII**T**LYL	0.915
485	**L**	482	488	IIT**L**YLG	0.917
486	**Y**	483	489	ITL**Y**LGA	0.917
487	**L**	484	490	TLY**L**GAV	0.924
488	**G**	485	491	LYL**G**AVV	0.925
489	**A**	486	492	YLG**A**VVQ	0.924
